# SMAC/Diablo controls proliferation of cancer cells by regulating phosphatidylethanolamine synthesis

**DOI:** 10.1002/1878-0261.12959

**Published:** 2021-05-04

**Authors:** Swaroop Kumar Pandey, Avijit Paul, Anna Shteinfer‐Kuzmine, Ran Zalk, Uwe Bunz, Varda Shoshan‐Barmatz

**Affiliations:** ^1^ Department of Life Sciences Ben‐Gurion University of the Negev Beer Sheva Israel; ^2^ National Institute for Biotechnology in the Negev Ben‐Gurion University of the Negev Beer Sheva Israel; ^3^ Ilse Katz Institute for Nanoscale Science & Technology Ben‐Gurion University of the Negev Beer Sheva Israel; ^4^ Organisch‐Chemisches Institut Ruprecht‐Karls‐Universität Heidelberg Germany; ^5^ Present address: Special Centre for Molecular Medicine Jawaharlal Nehru University New Delhi India

**Keywords:** cancer, mitochondria, phosphatidylethanolamine synthesis, phospholipids, PSD, SMAC/Diablo

## Abstract

SMAC/Diablo, a pro‐apoptotic protein, yet it is overexpressed in several cancer types. We have described a noncanonical function for SMAC/Diablo as a regulator of lipid synthesis during cancer cell proliferation and development. Here, we explore the molecular mechanism through which SMAC/Diablo regulates phospholipid synthesis. We showed that SMAC/Diablo directly interacts with mitochondrial phosphatidylserine decarboxylase (PSD) and inhibits its catalytic activity during synthesis of phosphatidylethanolamine (PE) from phosphatidylserine (PS). Unlike other phospholipids (PLs), PE is synthesized not only in the endoplasmic reticulum but also in mitochondria. As a result, PSD activity and mitochondrial PE levels were increased in the mitochondria of SMAC/Diablo‐deficient cancer cells, with the total amount of cellular PLs and phosphatidylcholine (PC) being lower as compared to SMAC‐expressing cancer cells. Moreover, in the absence of SMAC/Diablo, PSD inhibited cancer cell proliferation by catalysing the overproduction of mitochondrial PE and depleting the cellular levels of PC, PE and PS. Additionally, we demonstrated that both SMAC/Diablo and PSD colocalization in the nucleus resulted in increased levels of nuclear PE, that acts as a signalling molecule in regulating several nuclear activities. By using a peptide array composed of 768‐peptides derived from 11 SMAC‐interacting proteins, we identified six nuclear proteins ARNT, BIRC2, MAML2, NR4A1, BIRC5 and HTRA2 Five of them also interacted with PSD through motifs that are not involved in SMAC binding. Synthetic peptides carrying the PSD‐interacting motifs of these proteins could bind purified PSD and inhibit the PSD catalytic activity. When targeted specifically to the mitochondria or the nucleus, these synthetic peptides inhibited cancer cell proliferation. To our knowledge, these are the first reported inhibitors of PSD acting also as inhibitors of cancer cell proliferation. Altogether, we demonstrated that phospholipid metabolism and PE synthesis regulated by the SMAC‐PSD interaction are essential for cancer cell proliferation and may be potentially targeted for treating cancer.

## Graphical abstract

Apart from its pro‐apoptotic function, SMAC/Diablo acts as a regulator of lipid synthesis during cancer cell proliferation and development. We showed that SMAC/Diablo directly interacts with mitochondrial phosphatidylserine decarboxylase (mPSD) and inhibits its catalytic activity of phosphatidylethanolamine (PE) synthesis from phosphatidylserine (PS). Depletion of mitochondrial SMAC/Diablo activated mPSD, increased PE production and inhibited cancer cell proliferation. We concluded that SMAC‐PSD interactions are essential for carcinogenesis and may be potentially targeted for treating cancer.

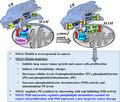

**Abbreviations**
ARNT: aryl hydrocarbon receptor nuclear translocator
BIRC2: baculoviral IAP repeat‐containing 2
ER: endoplasmic reticulum
HTRA2: high‐temperature requirement *protein* A2
IF: immunofluorescence
IHC: immunohistochemistry
IMS: intermembrane space
IP3R: 1,4,5‐trisphosphate receptor
KO: knock out
MAM: mitochondria‐associated membranes
MAML2: mastermind‐like transcriptional coactivator 2
MST: microscale thermophoresis
OMM: outer mitochondrial membrane
OPA: ophiobolin A
PC: phosphatidylcholine
PE: phosphatidylethanolamine
PLA: proximity ligation assay
PLs: phospholipids
PS: phosphatidylserine
PSD: phosphatidylserine decarboxylase
SMAC/Diablo: second mitochondria‐derived activator of caspase/direct inhibitor of apoptosis‐binding protein with low pI
TRAF2: TNF receptor‐associated factor 2
TTs: treated tumours
VDAC1: voltage‐dependent anion channel 1

## 1 Introduction

Alteration of tumour metabolism, including lipid metabolism is involved in carcinogenesis with cancer cells also, demonstrates a high dependence on lipids [1]. Phospholipids (PLs) are not only essential building‐block components of cellular membranes, but act as regulators for various cellular functions, such as cell adhesion and migration, neurotransmission, signal transduction, vesicular trafficking, apoptosis, metabolism and post‐translational modifications [2].

Changes in composition, distribution and metabolism of PLs in cells, tissues and body fluids (blood, urine) are associated with cancer and other diseases [3]. Aberrant phospholipid metabolism in cancer has recently been established as a universal metabolic hallmark of cancer, and the phospholipid content was shown to increase with cell transformation and tumour progression [4]. Changes in PL profiles have been associated with cancer diagnosis and treatment [5] and specific lipids might be involved in the onset and evolution of cancer [6]. Phosphatidylethanolamine (PE), the focus of this study, is the second most abundant phospholipid on mammalian cellular membranes. Comprising ∼ 25% of mammalian PLs, it is found predominantly in the inner leaflet of the plasma membrane and enriched in mitochondrial inner membranes [7]. Besides functioning as a membrane structural element, PE participates in many important pathophysiological cellular processes [8]. PE translocation and redistribution occur during cell division and cell death, and it is important for membrane fusion and remodelling [7]. PE itself or PE‐derived ethanolamine is covalently linked to diverse proteins, including signalling proteins, and to LC3 as a prerequisite for autophagosome formation [7]. Recently, PE synthesized in mitochondria was found to be important for mitochondrial function [9]. PE is known to be elevated in several cancers for decades [10]. PE’s importance for appropriate cancer cell function is demonstrated in this study.

Given that fundamental differences exist between the cellular membranes of healthy cells and tumour cells, targeting lipids in cancer cells with novel therapies is a promising approach. Together with the diversity in PE biological roles, and being elevated in several cancers [10], PE represents a unique molecular target.

In mammalian cells, distinct pools of PE are synthesized in either mitochondria [11] or endoplasmic reticulum (ER) membranes [7]. Mitochondrial‐associated membranes (MAM) act as bridges between the mitochondria and ER, with versatile functions [12], including supporting mitochondrial function and the synthesis of neutral lipids, and PLs [13]. In the MAM, phosphotidylserine (PS) is transferred to mitochondria [14] and is converted to PE by the mitochondrial enzyme phosphotidylserine decarboxylase (PSD) [15]. Loss of PSD causes defects in mitochondrial morphology and function and disrupts electron transport chain complex formation [7]. PSD is an essential protein with its knock out (KO) being embryonic lethal [16].

The mitochondrial intermembrane space (IMS) protein, SMAC/Diablo (second mitochondria‐derived activator of caspase/direct inhibitor of apoptosis‐binding protein with low pI), is a pro‐apoptotic [17, 18]. Recently, by addressing the unexpected SMAC overexpression in cancer cells [19, 20], despite its pro‐apoptotic activity, we identified a new nonapoptotic function associated with regulating lipid synthesis essential for cancer growth and development [21]. We demonstrated that silencing SMAC/Diablo expression using human‐specific siRNA (si‐hSMAC) decreased PLs and phosphatidylcholine (PC) levels along with altered expression of enzymes associated with their synthesis. Additionally, SMAC/Diablo depletion resulted in a reduced vesicle formation, cancer cell proliferation and the growth of lung cancer cells‐derived tumours. Nuclear morphology, and the expression of genes associated with the cell membrane, exosomes and ER‐ and Golgi‐related proteins were also altered in SMAC/Diablo depleted cells. These multiple effects of SMAC depletion suggest that it possesses additional nonapoptotic functions associated with regulating phospholipid synthesis and essential for cancer growth and development [21].

In this study, we show that the SMAC nonapoptotic new function is regulating PE synthesis by inhibiting PSD activity via direct interaction. SMAC downregulation resulting in PSD overproducing mitochondrial PE, leading to cell depletion of PC and PS and inhibition of cancer cell proliferation. Furthermore, we developed several peptides that bind to purified PSD and inhibit its activity, and as cell‐penetrating peptides targeted to the mitochondria or the nucleus, they inhibit cell proliferation. Thus, we present PSD with an essential function in cancer cells and as a new target for cancer therapy.

## 2 Materials and methods

### 2.1 Materials

Fluorescein isothiocyanate (FITC), hematoxylin and eosin, PS, PE, sulforhodamine B (SRB), Triton X‐100 and Tween‐20 were obtained from Sigma (St. Louis, MO, USA). Transfection agents JetPRIME and JetPEI were procured from PolyPlus transfection (Illkirch, France). Paraformaldehyde was obtained from Emsdiasum (Hatfield, PA, USA). Dulbecco’s modified Eagle’s medium (DMEM) media were obtained from Gibco (Grand Island, NY, USA). Normal goat serum (NGS) and the supplement FBS, l‐glutamine and penicillin/streptomycin were obtained from Biological Industries (BeitHaemek, Israel). 3,3‐Diaminobenzidine (DAB) was obtained from (Vector Laboratories ImmPact‐DAB, Burlingame, CA, USA). Primary antibodies and horseradish peroxidase (HRP)‐conjugated secondary antibodies were obtained from different sources; their source, and dilutions are detailed in Table S1. Distyrylbenzene‐bis‐aldehyde (DSB‐3) was synthesized in U. H. F. Bunz’s group.

Tissue array sections (US Biomax BCN601) were obtained from US Biomax Inc., Derwood, MD, USA). Human SMAC/Diablo‐specific siRNA (si‐hSMAC‐A) was synthesized by Genepharma (Suzhou, China). Customized 768‐peptide sequences derived from 11 SMAC‐interacting proteins were arrayed on glass slides, produced by INTAVIS (Intavis Peptide Services, Tübingen, Germany). Peptides were synthesized by GL Biochem (Shanghai, China) to > 95% purity.

### 2.2 Cell culture and transfection

A549 (human lung adenocarcinoma epithelial cell, known as glycolic cells), HEK‐293T (human embryonic kidney), H1563/CRL‐5875 (non‐small‐cell lung cancer adenocarcinoma, NCI‐H‐1563), H‐358 (bronchioalveolar carcinoma), HaCaT (spontaneously transformed aneuploid immortal human skin keratinocyte) and WI‐38 (Caucasian fibroblast‐like foetal lung cell) cell lines were purchased from the American Type Culture Collection (ATCC) (Manassas, VA, USA) and were maintained as per ATCC instructions. Cells were maintained in ATCC recommended medium at 37 °C in an incubator with 5% CO_2_. Cell lines were routinely tested for mycoplasma contamination. For SMAC overexpression, cells were seeded (200 000 cells/well) on 6‐well culture plates and allowed to adhere overnight. These adhered cells were transfected with PVMV3.1‐SMAC or empty vector using the JetPRIME transfection reagent (Illkirch, France), according to the manufacturers’ instructions. Medium was changed 6 h post‐transfection, and cells were harvested 48 h post‐transfection. Human SMAC/Diablo‐specific siRNA (si‐hSMAC‐A) sequences were as follows:

Sense 5′AAGCGGUGUUUCUCAGAATTGtt3′ and antisense 5′AACAAUUCUGAGAAACCCG Ctt3′. Cells were seeded (150 000 cells/well, 6‐well plate), and 24 h later, were transfected with 10–100 nm si‐NT or si‐hSMAC‐A using the JetPRIME transfection reagent, according to the manufacturer’s instructions.

### 2.3 Mouse xenograft experiment

Male Hsd:Athymic ' Nude‐Foxn1nu mice (6–8 weeks old) were obtained from Envigo and were kept in the university animal facility in temperature‐controlled rooms and provided with water and food pellets *ad libitum*. All procedures involving mice were approved by the Ben‐Gurion Institutional Animal Care and Use Committee.

Lung cancer A549 (3 × 10**^6^**) cells were implanted subcutaneously on the dorsal flanks. Tumour growth was recorded using digital calibre, and volumes were calculated using, volume = X^2^ × Y/2, where X and Y are the short and long tumour dimensions, respectively. Mice were divided randomly into three groups once the average tumour volume reached ˜ 80 mm^3^ and were treated subcutaneously with si‐NT or si‐hSMAC‐A mixed with JetPEI reagent (350 or 700 nm final concentration) three times a week. Mice were sacrificed at the end of experiment, and the tumours were excised and processed for immunohistochemistry (IHC) or frozen in liquid nitrogen for immunoblotting and RNA isolation.

### 2.4 CRISPR/Cas9 SMAC and PSD knockout

SMAC CRISPR/Cas9 knockout plasmid with GFP marker (sc‐402009) was purchased from Santa Cruz Biotechnology (Dallas, TX, USA). A549 and HEK‐293T cells were seeded in 6‐well cell culture plates (200 000 cells/well) and allowed to attach overnight. Cells were transfected with SMAC CRISPR/Cas9 knockout plasmid as per manufacturers’ instructions using JetPRIME transfection reagent. GFP‐positive cells were sorted using FACS (SY3200 cell sorter—Synergy) and plated in 96‐well plates (one cell/well). Cells grew for 10 days, and each colony was transferred to a separate well of 12‐well cell culture plates. Individual colonies having SMAC‐KO were selected for maintenance after immunoblotting for SMAC, and a single SMAC‐knockout colony was used in further experiments. CRISPR/Cas9‐mediated knockout of PSD was done in A549, HEK‐293T and NCI‐H1563 (H1563/CRL‐5875) cells by transfecting PSD CRISPR/Cas9 knockout plasmid with GFP marker (sc‐404398) as described for SMAC knockout, but no cells survived.

### 2.5 Peptide array

Customized 768‐peptide sequences derived from 11 SMAC‐interacting proteins produced by INTAVIS Peptide Services (GmbH & Co. KG, Tübingen, Germany**)** were arrayed on a glass slide. Peptide arrays were blotted with SMAC, PSD or peptides interacting with PSD using anti‐SMAC or anti‐PSD antibodies. The interaction of purified SMAC or PSD with glass‐bound peptide arrays was assessed by slide washing (three times, 10 min each) with Tris‐buffered saline (150 mm NaCl, 50 mm Tris/HCl, pH 7.4), followed by overnight incubation with blocking buffer (Tris‐buffered saline containing low‐fat dry milk, 2.5%, w/v). The slides were then incubated for 4 h or overnight with purified SMAC (0.8 µm) with and without preincubation with PSD (0.3 µm). Similarly, slides were incubated with PSD (0.15 µm) with or without preincubation with the indicated peptide (20 μm) in blocking buffer at room temperature. After extensive slide washing with Tris‐buffered saline containing 0.05% Tween‐20, SMAC or PSD binding was detected using anti‐SMAC or anti‐PSD antibodies and HRP‐conjugated anti‐rabbit or anti‐mouse IgG as a secondary antibody. The blots were developed using EZ‐ECL (Biological Industries), according to the manufacturer’s instructions.

### 2.6 Immunohistochemistry (IHC) and Immunofluorescence (IF)

Formalin‐fixed and paraffin‐embedded tumour sections were deparaffinized using xylene and a series of ethanol treatments. Sections were then incubated with 3% H_2_O_2_ for 10 min to block endogenous peroxidase activity. Antigen retrieval was done in 0.01 m citrate buffer (pH 6.0) at 95–98 °C for 30 min and washed with PBST (0.1% Tween‐20). In order to reduce nonspecific binding, sections were incubated in 10% NGS for 2 h and then incubated with primary antibodies (Table S1) overnight at 4 °C. For IHC, after washing with PBST, sections were incubated for 2 h at room temperature with HRP‐conjugated secondary antibodies, washed well with PBST and incubated with the substrate DAB Sections were washed with water, counterstained with haematoxylin and mounted with Vectashield mounting medium (Vector Laboratories). Sections were observed under a microscope (Leica DM2500, Wetzlar, Germany), and images were collected at 20× magnification with the same light intensity and exposure time. For IF, following overnight incubation with the primary antibodies, PBST‐washed sections were incubated with fluorescent‐tagged secondary antibodies for 2 h at room temperature in the dark. Following a wash with PBS, sections were incubated with DAPI for 15 min in the dark, washed, mounted with Vectashield mounting medium and viewed by confocal microscopy (Olympus 1X81, Tokyo, Japan).

IF staining of cells was performed in cells plated on sterile glass coverslips placed in 12‐well cell culture plates (30 000 cells/well) and incubated overnight in CO_2_ incubator washed with PBS and fixed with 4% paraformaldehyde. Cells were then subjected to IF staining as described above for tissue sections.

### 2.7 Protein extraction and immunoblot

Cells were harvested and washed twice with ice‐cold PBS, and the pellets were lysed on ice for 30 min in a lysis buffer (50 mm Tris/HCl, pH 7.5, 150 mm NaCl, 1 mm EDTA, 1.5 mm MgCl_2_, 10% glycerol, 1% Triton X‐100), freshly supplemented with a protease inhibitor cocktail (Calbiochem) and centrifuged (10 min, 12 000 ***g***). The protein concentration of the supernatant was determined, and cells were stored at −80 °C until used for gel electrophoresis and immunoblotting. Protein samples (10–20 μg) were subjected to SDS‐PAGE and immunoblotting, using the selected primary antibodies, followed by incubation with HRP‐conjugated secondary antibodies. HRP activity was determined using EZ‐ECL (Biological Industries). Band intensity was quantified using FUSION‐FX (Vilber Lourmat, Collégien, France).

### 2.8 Sulforhodamine B (SRB) cell proliferation assay

Cells were seeded in 96‐well cell culture plates (8000/well) and allowed to grow for 48 h. After washing with PBS, cells were fixed with 50% trichloroacetic acid and stained with SRB for 20 min. Excess SRB was removed, and cells were washed with 1% acetic acid. SRB extraction was done using 100 mm Tris‐base, and absorbance at 510 nm was determined using an Infinite M1000 plate reader (Tecan, Männedorf, Switzerland).

### 2.9 Cytoplasmic and nuclear protein fractionation

A549 lung cancer cells were trypsinized and centrifuged at 600 ***g*** for 5 min. Cell pellet was washed with PBS centrifuged at 600 ***g*** for 5 min, and cell pellet (2 × 10^6^ cells) was subjected to nuclear/cytosol fractionation using a Nuclear/Cytosol Fractionation Kit (K266‐25; BioVision, Milpitas, CA, USA), according to the manufacturer’s instructions. Following centrifugation at 6000 ***g*** for 10 min, the obtained supernatant (cytosolic fraction) and pellet (nuclear fraction) were resuspended in the original volume. Samples were subjected to immunoblotting using anti‐SMAC, anti‐PSD, anti‐ATP synthase 5a (mitochondria), anti‐GAPDH (cytosol) and anti‐H4 (nucleus) antibodies.

### 2.10 Preparation of mitochondria‐free and mitochondria‐enriched fractions

A549 and A549‐SMAC‐KO cells (1.7 × 10^8^) were trypsinized and centrifuged at 600 ***g*** for 5 min, and cell pellet was washed with PBS and centrifuged at 600 ***g*** for 5 min. Cell pellet was resuspended in 2.4 mL buffer (10 mm NaCl, 1.5 mm MgCl_2_ and 10 mm Tris, pH 7.5), allowing for cell swelling for 10 min on ice. Cell suspensions were transferred to a glass homogenizer and subjected to homogenization using a tight pestle for 10 strokes. Six millilitres of mitochondrial suspension buffer (210 mm mannitol, 70 mm sucrose, 5 mm Tris/HCl, pH 7.5, 1 mm EDTA, pH 7.5) was mixed, and an additional 6 mL of the buffer was added to the suspension. Aliquots of the homogenate were kept (total extract), and the rest was centrifuged at 1300 ***g*** for 5 min and the supernatant was kept. This treatment was repeated twice, and the combined supernatant was centrifuged at 17 000 ***g*** for 10 min to obtain a pellet (mitochondria) and supernatant (mitochondria‐free fraction). The pellet was resuspended in mitochondrial suspension buffer, and the protein amount was determined in all fractions.

### 2.11 Determination of total phospholipids, PC and PE, and PSD activity

Total lipids were extracted from the SMAC‐KO and wild‐type A549/HEK 293T cells by the Folch method [22]. Briefly, a cell suspension (2 × 10^7^ cells/mL) was made in CHCl_3_/MeOH (2 : 1, v/v) sonicated for 30 s. Water (116 μL·mL^−1^) was added to the suspension and shaken for 2 h at room temperature at 300 r.p.m. The aqueous suspension was heated for 10 min at 60 °C and stored at 4 °C for 3 h. Undissolved material was filtered through a 0.45‐μm syringe filter. Solvent was evaporated under nitrogen, and residues were re‐dissolved in CHCl_3_/MeOH for lipid analysis.

Total phospholipid content of the cells was determined calorimetrically at 488 nm based on the formation of a complex between the PLs and ammonium ferrothiocyanate. PC level was also determined by calorimetric analysis at 316 nm using ammonium thiocyanatocobaltate reagent, as described previously [21].

PE content in the lipid samples was measured as described previously [23]. Briefly, solvent was evaporated from the lipid samples under nitrogen gas, and samples were resuspended in 0.8 mm Triton X‐100. Sample aliquots were added to 96‐well plates containing (50 µL) reaction mixture (80 mm NaCl, 2 mm K_2_HPO4, pH 7.4) and of borate buffer (12.5 µL) (100 mm boric acid, 75 mm NaCl, 25 mm sodium tetraborate, pH 9). After mixing, 12.5 µL of 100 µm DSB‐3, in 10 mm K_2_HPO_4_ (pH 7.4), was then added and incubated for 2 h in the dark with shaking (100 r.p.m.). Fluorescence was measured using excitation λ_ex_ = 403 nm and emission λ_em_ = 508 nm. A calibration curve was obtained using PE (Biovision) as a standard.

To determine PSD activity, purified enzyme (0.2–0.4 μm) or crude cell lysate (0.25–0.5 μg of protein) was incubated with PS (50 μm) for 45 min at 30 °C in the dark with shaking under the conditions described above for PE determination. PSD activity was measured by determining the PE produced as described above, except that the signal obtained in the absence of substrate PS (representing endogenous PE) was subtracted from the signal obtained in the presence of PS.

### 2.12 DSB‐3 PE cell staining

A549 and A549‐SMAC‐KO cells were seeded on sterile cover slips in 24‐well cell culture plates (75 000 cells/well) and allowed to adhere for 24 h. MitoTracker® (250 nm in DMEM + 10% FBS) was added, and plates were incubated in CO_2_ incubator for 30 min at 37 °C. Cells were washed with PBS and incubated with DSB3 (10 μm in reaction buffer; 40 mm NaCl, 1 mm KPO4, pH 7.4) in CO_2_ incubator for 2 h. Cells were washed with PBS, fixed with 4% PFA for 20 min at room temperature, washed with PBS and stained with DAPI for 20 min at room temperature. Cover slips were washed with PBS and mounted with Vectashield, and imaging was done using confocal microscopy (Olympus 1X81).

### 2.13 Proximity ligation assay (PLA)

PLA was conducted as previously described [24] using an *in␣situ* detection reagent red kit (DuoLink InSitu PLA Probe kit; Sigma‐Aldrich) according to the manufacturer’s protocol. VDAC1‐IP3R and SMAC‐PSD were analysed using PLA [24]. Briefly, for tumour section, formalin‐fixed and paraffin‐embedded tumour sections (5‐μm thick) were deparaffinized and then permeabilized using 0.3% Triton X‐100 in PBS for 30 min, followed by blocking with Duolink® Blocking Solution for 1 h. Sections were incubated overnight with anti‐VDAC1 (rabbit 1 : 750) and anti‐IP_3_R (mouse, 1 : 750), washed twice with TBS‐Tween (0.01%) and then incubated for 1 h with the secondary anti‐mouse and anti‐rabbit antibodies (PLA probe MINUS and PLUS) that were conjugated to complementary oligonucleotide extensions, and ligation and amplification steps were performed per manufacturer’s instructions, and Texas red‐labelled oligonucleotide probes were detected.

For cells in culture, A549 cells and A549‐SMAC‐KO cells were seeded on 35‐mm coverslips cultured to 60% confluency, washed with PBS and fixed for 10 min at RT with fresh 4% formaldehyde solution in PBS, permeabilized with 0.1% Triton X‐100 in PBS for 15 min at RT, washed once with PBS and blocked for 30 min at 37 °C. Blocking was carried out using Duolink Blocking Solution for 1 h. For PLA for SMAC and PSD close association, anti‐SMAC (rabbit, 1 : 750) and anti‐PSD (mouse, 1: 750) were used followed by incubation with the secondary antibodies conjugated to the complementary oligonucleotide. Ligation and amplification steps were performed as described above for tumour sections. Cells were stained with DAPI and then mounted with round coverslips using aqueous mounting media with DAPI. Preparations were mounted in Vectashield mounting medium (Vector Laboratories). Images were collected using a confocal microscope (Olympus IX81). Quantitation of protein levels, as reflected in the staining intensity, was analysed in the whole area of the sections using image j software (Sun Microsystems, Inc, CA, USA).

### 2.14 Purified PSD and SMAC

Human recombinant SMAC (10339‐H08E) was produced by Sino Biologicals (Wayne, PA, USA). Purified recombinant protein (full length) of human phosphatidylserine decarboxylase (PSD), with N‐terminal His tag, expressed in *E.␣coli* was obtained from Acris‐OriGene.

### 2.15 Microscale Thermophoresis (MST)

MST analysis was performed using a NanoTemper Monolith NT.115 apparatus as described earlier [25]. Briefly, purified SMAC purchased from Sino Biologicals was fluorescently labelled using a NanoTemper Protein labelling kit BLUE (L001; NanoTemper Technologies, München, Germany). A constant concentration of SMAC was incubated with several concentrations of purified PSD in 10 mm Tris buffer, pH 7.4. After a 30‐min incubation, 8 µL of the samples was loaded into a glass capillary (Monolith NT Capillaries), and thermophoresis was analysed (LED 20%, IR laser 20%).

### 2.16 Cell penetration of fluorescein isothiocyanate (FITC)‐labelled peptides

To fluorescently label the CPP‐peptides (5 mm), they were incubated with 50 µm FITC for 30 min in 10 mm Tricine buffer, pH 8.7, at 37 °C. Unreacted reagent was removed by dialysis using membranes with a cut‐off of 1000 Da (DiaEASY Dialyzer Floating Rack; BioVision). A549 cells were incubated for 90 min with 5 µm FITC‐labelled peptide in serum‐free DMEM, washed with PBS, fixed with 4% formaldehyde and viewed under a confocal microscope (Olympus IX81).

### 2.17 Statistics

Data are represented as means ± SEM, and replicates are as indicated in figure legends. Numeric differences of interval level data between groups were compared using a two‐tailed Student's *t*‐test.

Statistical significance is reported at *P* < 0.05(*), *P* < 0.01(**) or *P* < 0.001(***).

## 3 Results

### 3.1 SMAC/Diablo depletion in tumours or cells in culture‐inhibited cell proliferation

**Fig. 1:**
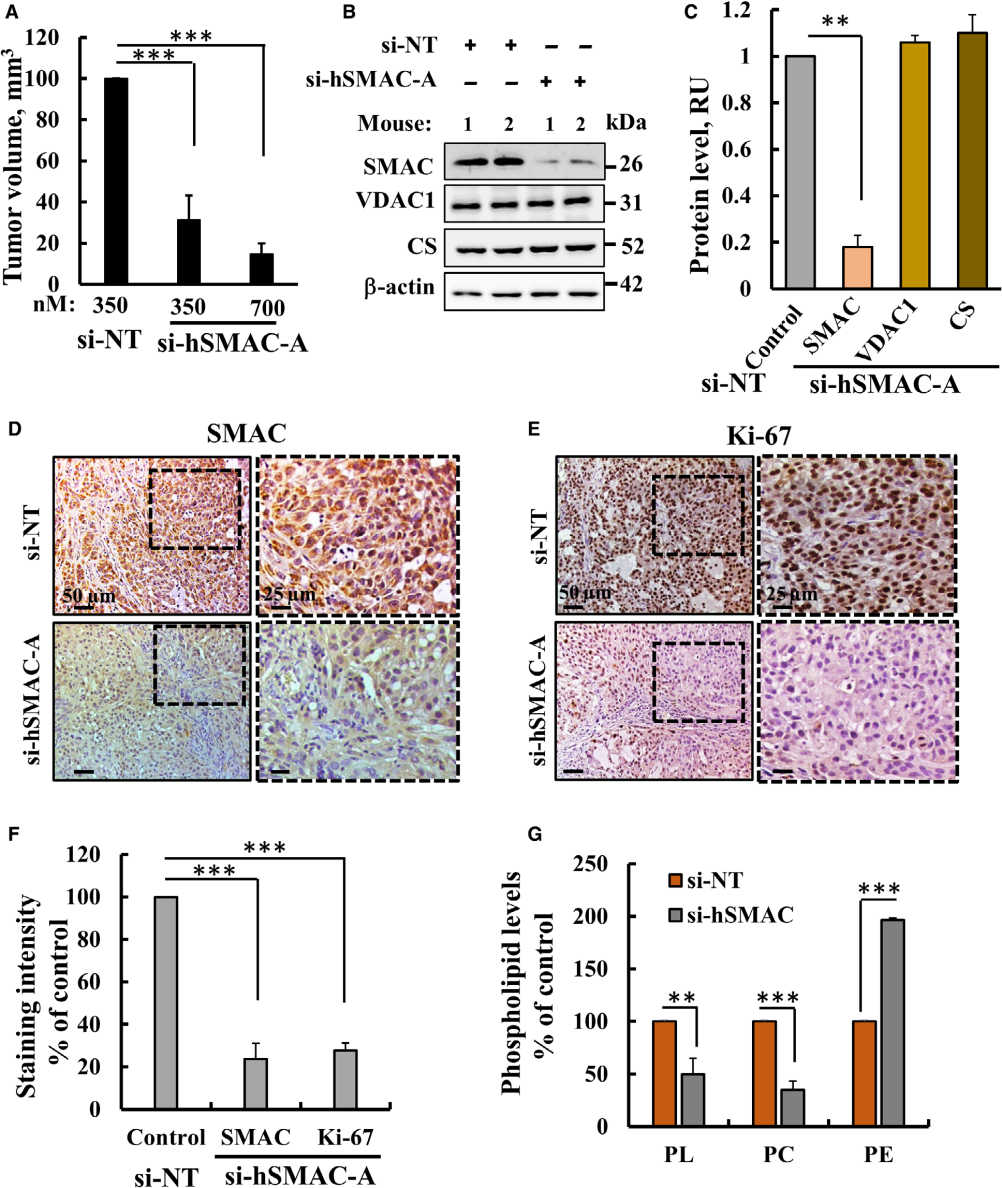
si‐hSMAC‐A treatment attenuates A549 lung cancer tumour xenograft growth and altered phospholipid levels. (A) Tumour growth of the A549 cell xenograft in immunodeficient 6‐week‐old male athymic nude mice. Mice were treated twice a week intratumourally with si‐hSMAC‐A (350 and 700 nm) or with nontargeted si‐RNA (si‐NT, 350 nm) (*n* = 8 mice/group). Treatment was initiated when the average tumour volume in each group reached ˜ 50 mm^3^. Results are presented as the mean tumour volume ± SEM. (B,C) Immunoblot (B) and quantitative analysis (C) of SMAC, VDAC1 and citrate synthase (CS) in si‐hSMAC‐TTs and si‐NT‐TTs samples (*n* = 6 mice/group). (D‐F) IHC stained of si‐hSMAC‐ or si‐NT‐TTs sections for SMAC (D), Ki‐67 (E), and their quantitative analysis (F) (*n* = 6 mice/group). Scale bars represent 50, 25 or 15 µm as indicated. (G) PL, PC and PE analysis was carried out as described in Materials and methods section (*n* = 6 mice per group). Results are the means ± SEM. *P* values were calculated using two‐sided Student's *t*‐test, **P ≤ 0.01; ***P ≤ 0.001.

We recently demonstrated that silencing SMAC/Diablo expression using specific siRNA (si‐hSMAC‐A) in subcutaneous xenografts of lung cancer A549 cells in mice reduced tumour growth [21]. Similarly, tumour volume was reduced by 50% and 85% when treating mice with si‐hSMAC‐A at 350 and 700 nm, respectively, relative to the nontargeting si‐RNA (si‐NT)‐treated tumours (TTs) (Fig. 1A). Immunoblotting (Fig. 1B,C) and IHC (Fig. 1D‐F) staining demonstrated that si‐NT‐TTs expressing high levels of SMAC/Diablo, that was decreased by 75% in si‐hSMAC‐A‐TTs (Fig. 1D,F). The decrease in tumour volume is resulted from cell proliferation inhibition as the expression of Ki‐67, a cell proliferation marker, was highly reduced in the si‐hSMAC‐A‐TTs (Fig. 1E,F). No cell death was detected in either si‐NT‐TTs or si‐hSMAC‐A‐TTs, as revealed by TUNEL staining (data not shown) [21].

The amounts of total PLs, PC and PE were analysed using the PE probe DSB3 [23]. Relative to si‐NT‐TTs, in si‐hSMAC‐A‐TTs, the levels of PLs and PC were decreased by 50% and 65%, respectively, while the levels of PE were increased by 200% (Fig. 1G).

**Fig. 2:**
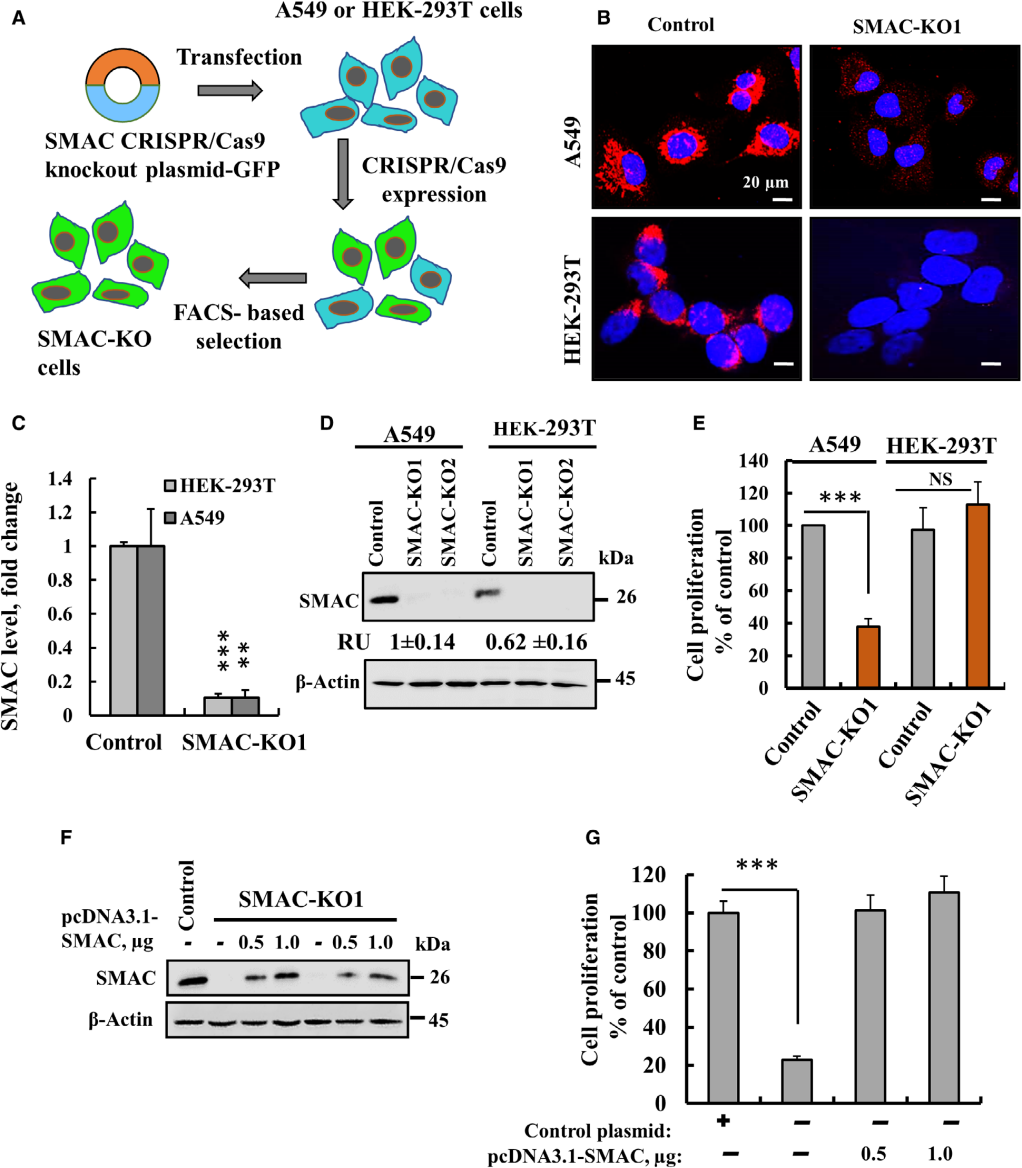
SMAC knockout in A549 cells by Crisper/Cas inhibits cell proliferation that could be restored upon SMAC re‐expression. (A) Schematic presentation of CRISPR/Cas9 mediates knockout. (B‐D) Representative IF images (scale bars = 20 µm) (B) their quantification (C) of SMAC in control and CRISPR/Cas9‐generated SMAC‐deficient A549 cells and HEK‐293T cells, and immunoblotting (D) stained with anti‐SMAC antibodies. (E) Cell proliferation in SMAC knockout A549 and HEK‐293T cells (*n*‐4) as analysed using the SRB method (*n* = 4). (F,G) A549 cells expressing SMAC were transfected with control plasmid, and SMAC knockout A549 cells were transfected with plasmid pCDNA3.1 (0.5 or 1 µg DNA) encoding full‐length SMAC. After 48 h cells were analysed by immunoblotting for SMAC expression (F) and cell proliferation (*n* = 4) (G). Results are the means ± SEM, *P* values were calculated using two‐sided Student's *t*‐test, **P ≤ 0.01; ***P ≤ 0.001; NS, nonsignificant.

To validate the results obtained with the lung cancer xenograft, showing that their proliferation is highly dependent on SMAC and study SMAC mode of action, we generated CRISPR/Cas9‐mediated SMAC knockout (SMAC‐KO) A549 lung cancer (A549:*SMAC*Δ/Δ) and HEK‐T‐293 (HEK:*SMAC*Δ/Δ) noncancerous cell lines )Fig. 2A). SMAC depletion in the A549:*SMAC*Δ/Δ and HEK:*SMAC*Δ/Δ cells was validated by immunofluorescence (IF) (Fig. 2B,C) and immunoblotting using anti‐SMAC antibodies (Fig. 2D). The lung cancer A549:*SMAC*Δ/Δ, but not HEK:*SMAC*Δ/Δ cells showed decreased cell proliferation (65%) as analysed using the sulforhodamine B (SRB) assay (Fig. 2E).

To demonstrate that SMAC depletion is responsible for the inhibited cell proliferation, SMAC was re‐expressed in the A549:*SMAC*Δ/Δ cells using a SMAC‐encoding plasmid. Re‐expression of SMAC in these cells (Fig. 2F) restored cell proliferation (Fig. 2G), suggesting that the inhibited cell proliferation is directly related to SMAC cell depletion.

### 3.2 SMAC depletion in lung tumours increased ER‐mitochondria contact sites (MAM)

**Fig. 3:**
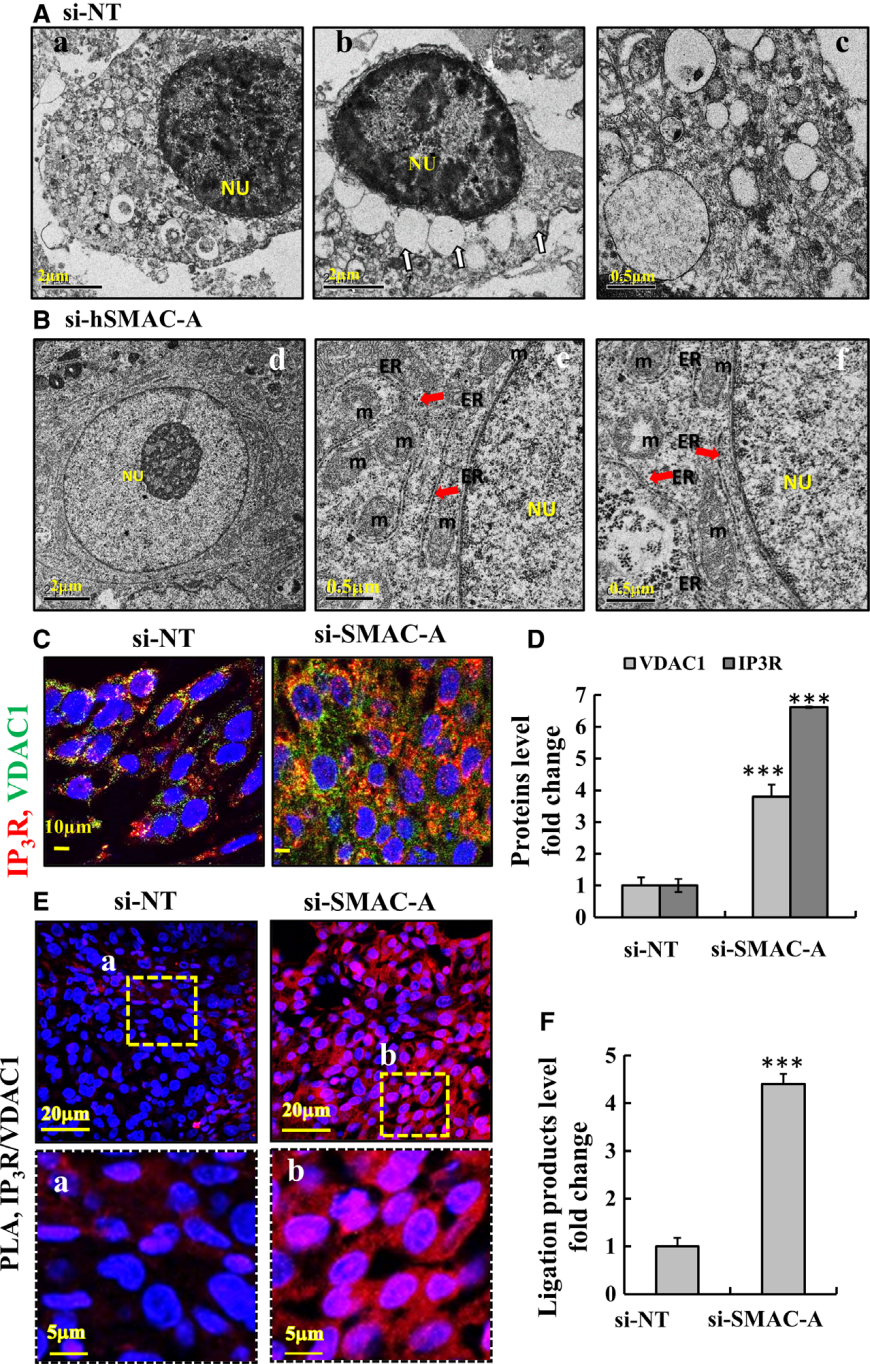
Subcellular morphological alterations including ER‐mitochondria contact sites, as induced by reduction of SMAC levels. (A,B) Representative transmission electron micrographs of sections from si‐NT‐TTs (A) and si‐hSMAC‐A‐TTs (B) from A549 xenografts carried out as described previously [21]. Various membrane organelles as intracellular vesicles containing surfactant‐accumulating lamellar bodies MAM‐like structures (ER‐associated mitochondria) are seen in the si‐NT‐TTs (Aa‐c), but not in si‐hSMAC‐A‐TTs, showing enrichment in the mitochondria (m), ER and nucleus (Nu). White (b) and red (e,f) arrows point to lamellar bodies and ER, respectively. Mitochondria number in si‐NT‐TTs and si‐hSMAC‐A‐TTs per EM section was 1‐3 and 8‐15, respectively. Scale bars represent 2 or 0.5 µm as indicated. (C,D) IF staining for IP_3_R (red) and VDAC1 (green) in si‐NT‐TTs and si‐hSMAC‐A‐TTs sections. Images were captured by confocal microscope (*n* = 5, scale bar represents 10 µm). (C) and subjected to quantitative analysis (*n* = 5) (D). (E,F) si‐NT‐TTs and si‐hSMAC‐TTs were subjected to *in‐situ* PLA to test for close association between VDAC1 (OMM) and IP_3_R (ER) (MAM) using specific antibodies. The ligation products appear in red; nuclei were DAPI‐stained. Images at high magnification (Ea,b) (scale bars represent 20, 5 µm as indicated) and ligation product quantification (F) are also shown. Results are the means ± SEM, *P* values were calculated using two‐sided Student's *t*‐test, ***P ≤ 0.001.

Using transmission‐electron microscopy (TEM), major alterations in the subcellular ultrastructures of si‐hSMAC‐A‐TTs relative to that of si‐NT‐TTs were identified [21]. In si‐NT‐TTs, a huge number of intracellular vesicles of different sizes and densities, for instance the large vesicles containing surfactant‐accumulating lamellar bodies and others, were observed (Fig. 3A). Such vesicles were not observed in the si‐hSMAC‐A‐TTs (Fig. 3B). In addition, in si‐NT‐TTs, the nuclear DNA was darkly stained representing heterochromatin, while in si‐hSMAC‐A‐TTs, the nuclear DNA was found as euchromatin and not markedly stained (Fig. 3A,B).

As the main location of PL synthesis is mitochondria‐ER contact sites (MAM) [12], we looked for MAM‐like structures in the EM images. Interestingly, si‐hSMAC‐A‐TTs sections were highly enriched in mitochondria (m) surrounded by ER (Fig. 3B: e,f) that may represent MAMs.

To demonstrate possible increase in MAMs in the si‐hSMAC‐A‐TTs, tissue sections were IF stained for the ER protein 1,4,5‐trisphosphate receptor (IP3R) and for VDAC1 as outer mitochondrial membrane (OMM) protein using specific antibodies (Fig. 3C,D). The results of the IF staining and their quantitative analysis clearly indicate that both IP_3_R and VDAC1 levels in si‐hSMAC‐A‐TTs were highly increased relative to their staining in si‐NT‐TTs (Fig. 3C,D).

Next, to validate that the increase in ER surrounding mitochondria (Fig. 3B) represents MAM, *in‐situ* proximity ligation assay (PLA), enables visualizing protein–protein interactions [26] was performed (Fig. 3E,F), using anti‐VDAC1 and anti‐IP3R antibodies. The results show a strong signal (over fourfold) in the si‐hSMAC‐A‐TTs sections relative to that observed in si‐NT‐TTs, suggesting an increase in MAM (Fig. 3E,F). This is confirming the close proximity of VDAC1 at the mitochondria and IP_3_R at the ER, both components of the MAM [12]. No signal was obtained in PLA using SMAC and the inner mitochondria membrane (IMM) protein, ATP synthase subunit 5A antibodies (Fig. S1).

### 3.3 SMAC cell depletion in cancer cells regulates phospholipid synthesis via modulating the mitochondrial PSD

**Fig. 4:**
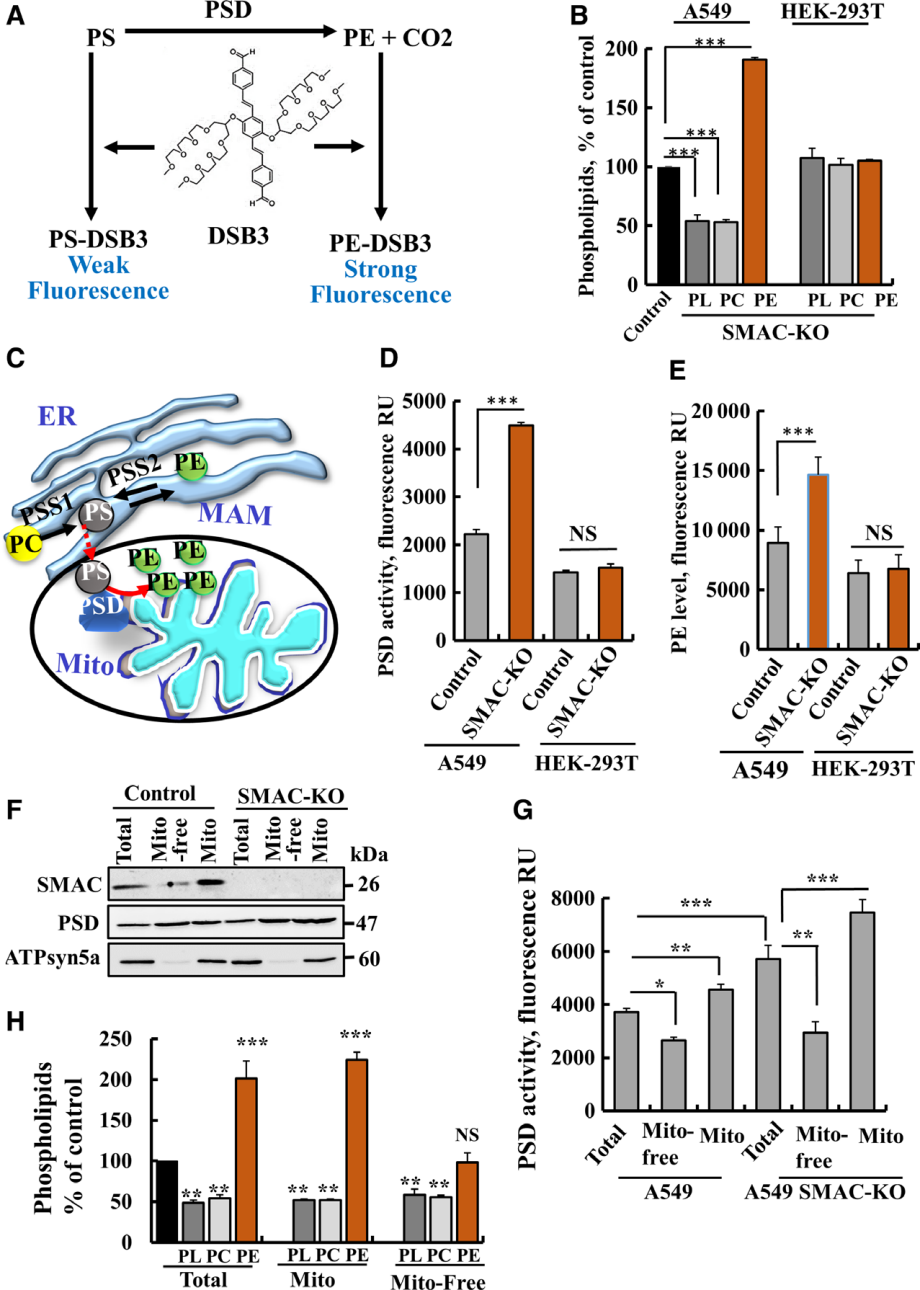
SMAC knockout in A549, but not in HEK‐293T cells reduces PL and PC, and increases PE. (A) Schematic presentation of PE reaction with DSB‐3 and obtained fluorescence signals used for PSD activity analysis. (B) PLs were extracted from the indicated cells and analysed for total PL, PC and PE levels as described in the Materials and methods section. (C) Schematic presentation of PS imported from the ER into the mitochondria at the MAM and its conversion to PE by PSD. (D,E) PSD activity (D) and PE levels (E) in control and SMAC‐KO, A549 and HEK‐293T cells analysed as described in the Materials and methods section. (F,G) Control and SMAC‐knockout A549 cells and their mitochondria‐free and mitochondria‐enriched subfractions (obtained as described in the Materials and methods section) were subjected to immunoblotting (F) or PSD activity assay (G). Total PL, PC and PE as analysed in SMAC‐KO A549 cell extract, mitochondria‐free and mitochondria‐enriched fractions relative to their levels in SMAC‐expressing A549 cells (H). Results are the mean ± SEM (*n* = 3); *P* values were calculated using two‐sided Student's *t*‐test, *P ≤ 0.05, **P ≤ 0.01; ***P ≤ 0.001, NS, nonsignificant.

SMAC‐expressing and SMAC‐KO A549 cells represent comparative models to investigate SMAC function in regulating phospholipid synthesis. The levels of PLs, PC and PE in the A549‐SMAC‐KO‐ and HEK‐293T‐KO cells relative to SMAC‐expressing cells were analysed. In the lung cancer A549‐SMAC‐KO cells, the levels of PLs and PC were reduced (twofold), while the level of PE (assayed by DSB3 [23], Fig. 4A) was increased twofold (Fig. 4B). HEK‐293T‐SMAC‐KO and SMAC‐expressing cells, however, showed similar levels of PL, PC and PE (Fig. 4B). Interestingly, the cell size of the A549‐SMAC‐KO was increased (Fig. S2A,B), which may suggest changes in membrane fluidity.

One of the key proteins in PE synthesis is PSD, a mitochondrial enzyme localized as SMAC in the IMS. PSD catalyses the formation of PE from PS transported from the ER to the mitochondria (Fig. 4C). Therefore, we asked whether the increase in PE obtained in the lung cancer A549 SMAC‐KO cells was due to increased PSD activity owing to the absence of SMAC. Accordingly, we analysed in A549‐SMAC‐KO cells the PSD activity and PE levels that were increased about twofold relative to SMAC‐expressing A549 cells, while HEK‐293T SMAC‐expressing or SMAC‐KO cells showed similar PSD activity (Fig. 4D) and PE levels (Fig. 4E). These results indicate that upon SMAC‐KO, PSD activity and PE levels were increased in cancer cells, but not in the tested nontumourigenic cell line. It should be indicated that HEK‐293 can be tumourigenic [27]. Next, we asked whether PE was increased in the mitochondria of SMAC‐KO cells because of the activation of PSD. PSD catalyses the production of PE from PS, produced in the ER from PC and PE by PS‐synthase 1 (PSS1) and PSS2, respectively [28] (Fig. 4C). Mitochondria‐enriched and mitochondria‐free fractions were obtained from control cells and SMAC‐KO A549 cells, as validated by the absence of the IMM protein, ATPsyn5a in mitochondria‐free fraction (Fig. 4F). SMAC is enriched in the control mitochondria fraction and as expected, lacking in the SMAC‐KO cells, while PSD is found in both mitochondria‐free and mitochondria‐enriched fractions (Fig. 4F). These fractions were analysed for PSD activity, showing increased activity in SMAC‐KO cell extract, as well as in the mitochondrial fraction derived from control and further increased in SMAC‐KO cells (Fig. 4G, Table S2).

The levels of PLs, PC and PE in the total PLs extract in these fractions were also analysed (Fig. 4H). As found for the si‐hSMAC‐TTs (Fig. 1G) and SMAC‐KO cells (Fig. 4B), the levels of PLs and PC were decreased about twofold, while PE level was over twofold higher in both the total cell phospholipid extract and in the mitochondria‐enriched fraction, but not in the mitochondria‐free fraction (Fig. 4H). The increase in PE levels in the SMAC‐KO cells (Fig. 4B) and tumours (Fig. 1G) suggests that upon SMAC depletion, PSD is activated, leading to increased PE production in the mitochondria at the expense of PC, PS, and PE in the ER.

### 3.4 PE‐specific probe DSB‐3 can be used to unravel PE levels in subcellular compartments

The PE‐specific probe DSB‐3, used to assay PE levels in solution, is used here, for the first time, to unravel PE levels in subcellular compartments in A‐549 cells, using confocal fluorescence imaging (Fig. S3). Even though DSB‐3 is a small molecule probe, and PE is present in most membranes, the results showed that PE was enriched in the mitochondria. The results clearly show green staining representing DSB‐3 labelled PE at the plasma membrane (white arrow, Fig. S3 a,c, Fig. S3B: d, Fig. S3C: a,c,d), and in other cell compartments, possibly ER. In addition, all green fluorescence was colocalized with MitoTracker (red colour), (Fig. S3A,C: b,c,e, Fig. S3D, orange colour blue arrows), suggesting that most PE is in the mitochondria, in agreement with the enrichment of PE in the mitochondrial fraction of SMAC‐KO cells (Fig. S3D). An apoptotic cell with membrane blebbing is also shown (Fig. S3C: e).

PE‐specific probes, duramycin and cinnamycin, that bind to the head group of PE were developed and used to follow PE on a cellular and tissue level, but not subcellular level [29]. These reagents affect membrane reorganization and permeabilization, eventually leading to cell death [30]. DSB‐3 on the other hand induces no cell death (data not shown) and, thus, may assist in unravelling PE levels and subcellular localization under different conditions.

### 3.5 SMAC and PSD expression levels in cancer and noncancerous cell lines and SMAC interaction with and inhibiting PSD activity

**Fig. 5:**
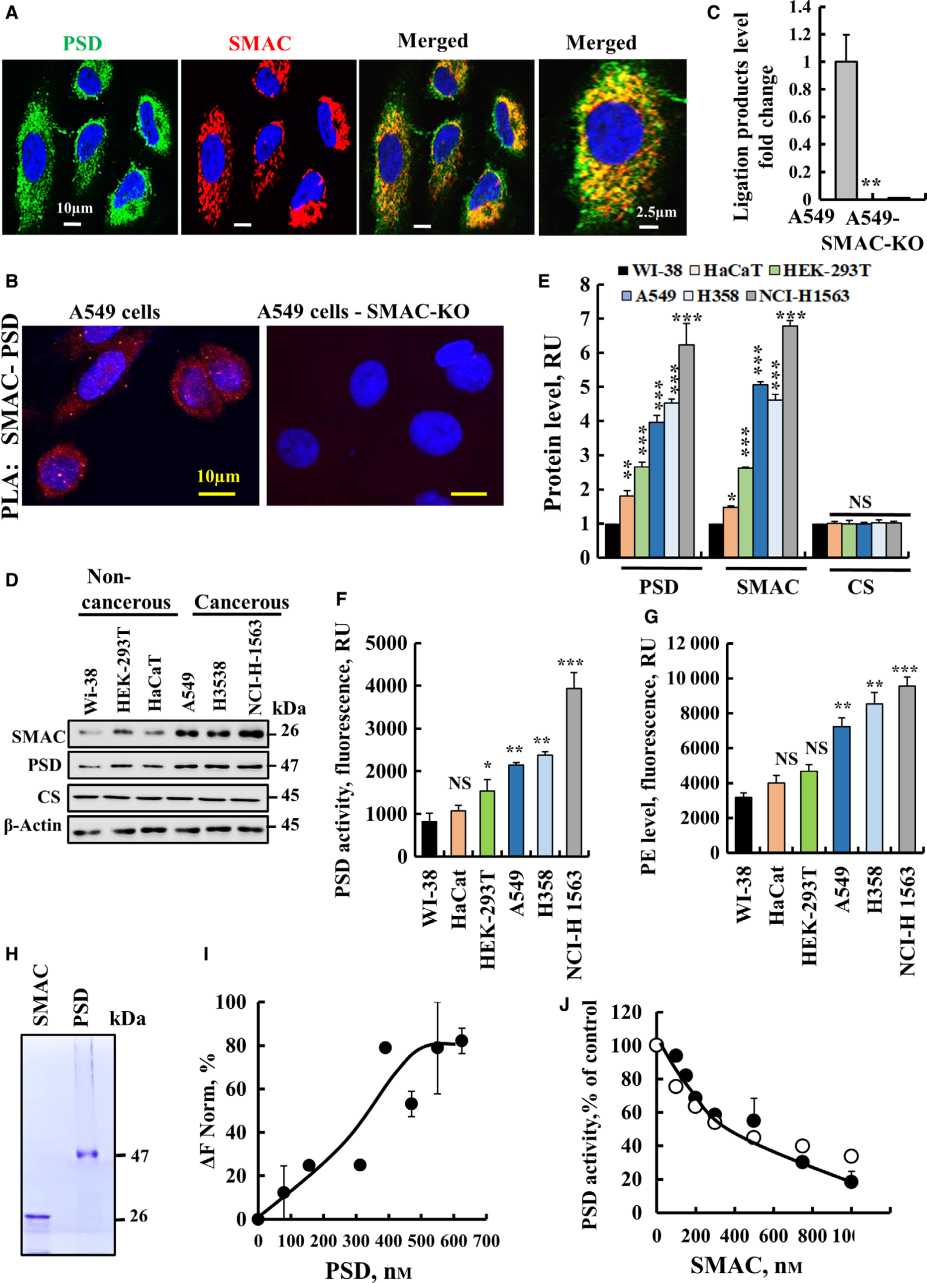
SMAC is colocalized with PSD, interacts with and negatively regulates PSD activity. (A) Representative IF images for SMAC and PSD colocalization in A549 cells. Scale bars represent 10, 2.5 µm, as indicated. (B,C) PLA assay showing direct interaction between SMAC and PSD in A549 cells (scale bar represent 10 µm). (B) and quantitative analysis (*n* = 3) of the ligation product (C) Results are the mean ± SEM (*n* = 3) *P* values were calculated using two‐sided Student's *t*‐test, **P ≤ 0.01. (D‐G) Immunoblot (D) and quantitative analysis cells (E) of PSD and SMAC expression levels relative to WI‐38 (*n* = 3), PSD activity (*n* = 6) (F) and PE levels (*n* = 6) (G) were analysed in the indicated cell lines. (H) Purified PSD and SMAC used in this study. (I) PSD interaction with SMAC analysed using the MST method (*n* = 4). Purified SMAC was fluorescently labelled with the NanoTemper BLUE protein‐labelling kit. SMAC (1 µm) was incubated with purified PISD (78–625 nm) for 30 min at 37 °C, then thermophoresis was measured as described in the Materials and methods section. *K*_d_ = 246 ± 29 nm (*n* = 3). (J) Inhibition of PSD activity by SMAC, as measured using the DSB‐3 method (*n* = 4). Results are the mean ± SEM. *P ≤ 0.05; **P ≤ 0.01; ***P ≤ 0.001; NS, nonsignificant.

SMAC and PSD are both located in the IMS, as also demonstrated here by IF staining (Fig. 5A). The close association of SMAC with PSD is demonstrated by *in‐situ* PLA assay, with a signal obtained in SMAC‐expressing, but not in SMAC‐KO A549 cells (Fig. 5B,C), indicating close proximity of the two proteins. PLA was previously used to demonstrate interaction between proteins such as Myc and Max in response IFN‐γ signalling [26].

The relationship between SMAC and PSD was evaluated by analysing their expression levels (Fig. 5D,E), PSD activity (Fig. 5F) and PE levels (Fig. 5G) in different cell lines, cancerous (A549, H3538, NCI‐H‐1563) and nontumourigenic cell lines (WI‐38, HEK‐293T, HaCat). The results indicate that both proteins were highly expressed (2‐ to 7‐fold) in cancerous cells relative to noncancerous cells, with no significant difference in the expression of the mitochondrial protein, citrate synthase (Fig. 5D,E).

Similar results were obtained when PSD activity (Fig. 5F) or PE levels (Fig. 5G) were analysed. The results show a correlation between SMAC and PSD expression level, PSD activity and PE levels with NCI‐H‐1563 showing the highest values.

The direct interaction of purified SMAC with purified PSD (Fig. 5H) was demonstrated using the microscale thermophoresis (MST) (Fig. 5I). PSD interacts with SMAC with a high affinity (*K*_d_ = 246 nm). SMAC interaction with PSD also is reflected in SMAC‐inhibiting PSD activity with IC_50_ of 400 nm (Fig. 5J), in agreement with the increase in PE levels in si‐hSMAC‐TTs (Fig. 1G) and in CRISPER/Cas9‐KO cells (Fig. 4E,H).

### 3.6 Identification of SMAC–PSD interaction site using peptide arrays composed of peptides derived from SMAC‐interacting proteins

**Fig. 6:**
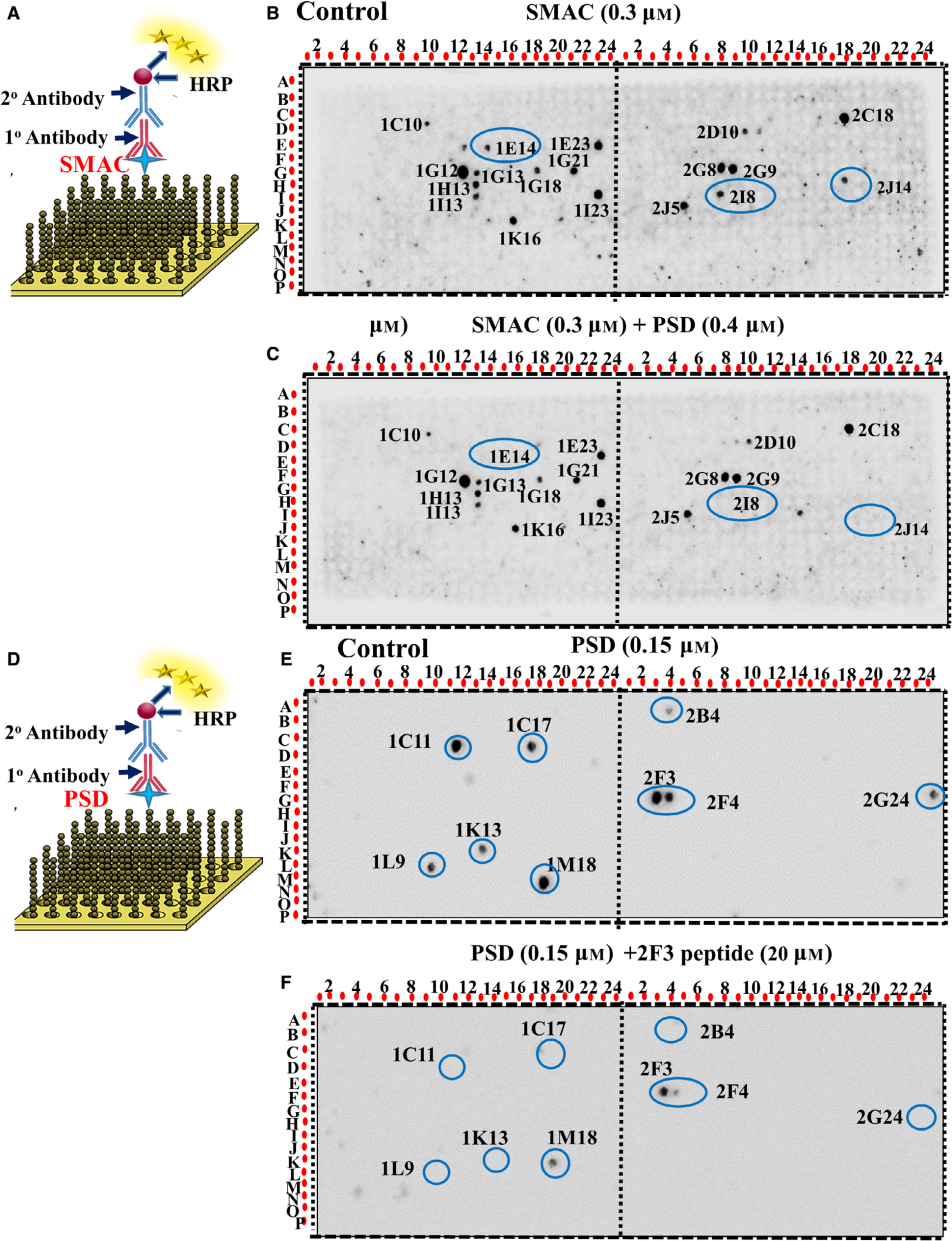
PSD‐ and SMAC‐interacting partners. (A) Schematic presentation of peptide array and detection of their interaction with SMAC. (B) Glass‐bound peptide array consisting of overlapping peptides derived from 15 SMAC‐interacting proteins were incubated overnight with purified SMAC (0.8 µm) and then blotted with anti‐SMAC antibodies (1 : 1000), followed by incubation with HRP‐conjugated anti‐mouse IgG and detection using a chemiluminescence kit. Dark spots represent binding of SMAC to peptides derived from SMAC‐interacting proteins. (C) SMAC (0.3 µm) was incubated with purified PSD (0.4 µm) and blotted as in (B). The peptide spots where PSD prevented interaction with SMAC (Table S1) are circled. (D) Schematic presentation of peptide array and detection of their interaction with PSD. (E) Peptide array was blotted with purified PSD (0.15 µm) and then with anti‐PSD antibodies as in (B). (F) PSD (0.15 µm) was preincubated with its interacting peptide representing spot 2F3 (20 µm), followed by array blotting with anti‐PSD antibodies as in (E). The peptide spots where 2F3 peptide prevented interaction with PSD are circled. Each presented peptide array represent 2–3 similar experiments.

To identify possible SMAC–PSD interaction site(s), we designed glass‐bound peptide arrays consisting of 768 overlapping peptides derived from 15 selected SMAC‐interacting proteins (reported and identified using the string link programme) and tested whether PSD interferes with SMAC interaction with any peptide in the array. The array was blotted with purified SMAC and then with anti‐SMAC antibodies (Fig. 6A), showing the binding of SMAC to 16 peptides derived from nine proteins (Fig. 6B, Table S3).

Preincubation of SMAC with purified PSD prevented SMAC interaction with three peptide spots: 1E14, a peptide derived from the protein BIRC2 (baculoviral IAP2 repeat‐containing 2); peptide 2I8, derived from MAML2 (mastermind like transcriptional coactivator 2 truncated poly Q); and peptide 2JI4, derived from ARNT (aryl hydrocarbon receptor nuclear translocator isoform 1, also known as HIF‐1β subunit) (Figs 6C and S4A, Table 1).

**Table 1:** Proteins identified as interacting with PSD or their interaction with SMAC/Diablo are prevented in the presence of PSD. Results are from Fig. 6, presenting the peptides derived from these proteins and, their position in the peptide array. Protein function and cellular localization are indicated.

Protein/function	Identified by blotting with PSD Spot No, Sequence	Identified by PSD inhibiting SMAC binding, Spot No, Sequence
HTRA2—Serine peptidase 2. Serine protease promotes cell death either by direct binding to IAPs proteins leading to increased caspase activity or a caspase‐independent and serine protease activity‐dependent mechanism. (Mitochondrion intermembrane space, nucleus [76])	1C11‐ALLTSGTSDPRA RVTYGTPSLWRL 1C17‐RTREASENSGT RSRAWLAVALGAGG	
BIRC2—Baculoviral IAP2 repeat. Acts as an E3 Ubiquitin–protein ligase. Regulator of NF‐kappa‐B signalling, apoptosis, cell proliferation, cell invasion, and metastasis. Modulates inflammatory signalling (cytosol, nucleus [32] and plasma membrane)	1F5‐MSTEEARFLTYH MWPLTFLSPSELA	1E14‐FQQLTCVLPI LDNLLKANVINKQEH
TRAF‐2 —TNF receptor‐associated factor 2. Regulates activation of NF‐κB and JNK, cell survival and apoptosis. Constituent of several E3 ubiquitin–protein ligase complexes regulates BIRC2, BIRC3, RIPK1 and TICAM1 protein levels by inhibiting their autoubiquitination. (Cytosol)	1K13‐EMEASTYDGF IWKISDFARKRQE	
MTFR1—Mitochondrial fission regulator 1. Promotes mitochondrial fission, and deficiency of Mtfr1 results in oxidative DNA damage (mitochondria)	2B4‐EMNSVKLRSVK RSEQDVKPKPVDAT	
MAML2—Mastermind‐like transcriptional coactivator 2 truncated poly Q. A transcriptional coactivator for NOTCH proteins. Promotes proliferative signalling during neurogenesis (Nucleus) [77]	2F3‐ALSTSSPIPSVPQ SQAQPQTGSGAS 2F4‐SVPQSQAQPQT GSGASRALPSWQEV 2G24‐KDQRRNVGN MQPTAQYSGSSTISL	2I8‐PNQSSRAFQGT DHSSDLAFDFLSQQ
ARNT—Aryl hydrocarbon receptor nuclear translocator isoform 1 (known as HIF‐1β). ARNT forms a complex with the ligand‐bound aryl hydrocarbon receptor. When bound, the ligand translocates from the cytosol to the nucleus involving its translocator (AREN). Identified as the beta subunit of a heterodimeric transcription factor, hypoxia‐inducible factor 1 (HIF1), and functions as a transcriptional regulator of the adaptive response to hypoxia. (Nucleus) [33]	2K24‐NHSQVVQPVTT TGPEHSKPLEKSDG	2J14‐DVDKLREQLS TSENALTGRILDLKT
NR4A1—Nuclear receptor subfamily 4 group A. Member of the steroid‐thyroid hormone–retinoid receptor superfamily. Acts as a nuclear transcription factor. Translocation of the protein from the nucleus to mitochondria induces apoptosis. (Nucleus, mitochondria) [78]	1L9‐KADGIMWLAKA CWSIQSEMPCIQAQ 1M18‐GVRTCEGCKGF FKRTVQKNAKYICL	

Using available structures for BIRC, MAML2 and ARNT proteins, the sequences interacting with PSD were localized on the protein surface (prepared using with UCSF [31]) thus, exposed to interaction with PSD (Fig. S5A). This may suggest that the interaction of PSD with SMAC prevented SMAC interaction with these three peptides, alternatively, PSD interacts with these peptides in the array and prevents SMAC binding to them.

To test this, we blotted the peptide array with purified PSD followed by anti‐PSD antibodies and found PSD interaction with nine peptides derived from seven different proteins (Figs 6D,E and S4, Table 1), the IMS protein HTRA2 (serine peptidase 2), TRAF‐2 (TNF receptor‐associated factor 2), MTFR1 (mitochondrial fission regulator 1), MAML2, ARNT and BIRC2. Interestingly, PSD prevented MAML2, ARNT and BIRC2 binding to SMAC (Fig. 6C, Table 1). However, PSD interacted with different peptides derived from those proteins than those it prevented SMAC binding to (Tables 1 and S3). Remarkably, these three proteins are located in the nucleus [32, 33]. SMAC and PSD presence also in the nucleus is presented below.

The peptides, 2F3, derived from the nuclear protein MAML2, and IC11, derived from IMS protein HTRA2 showing the highest interaction with PSD (as reflected in the spot intensity, Fig. 6E), were synthesized and when preincubated with PSD, they eliminate PSD interaction with most peptides (Figs 6F and S5B), pointing to PSD‐specific interaction.

**Fig. 7:**
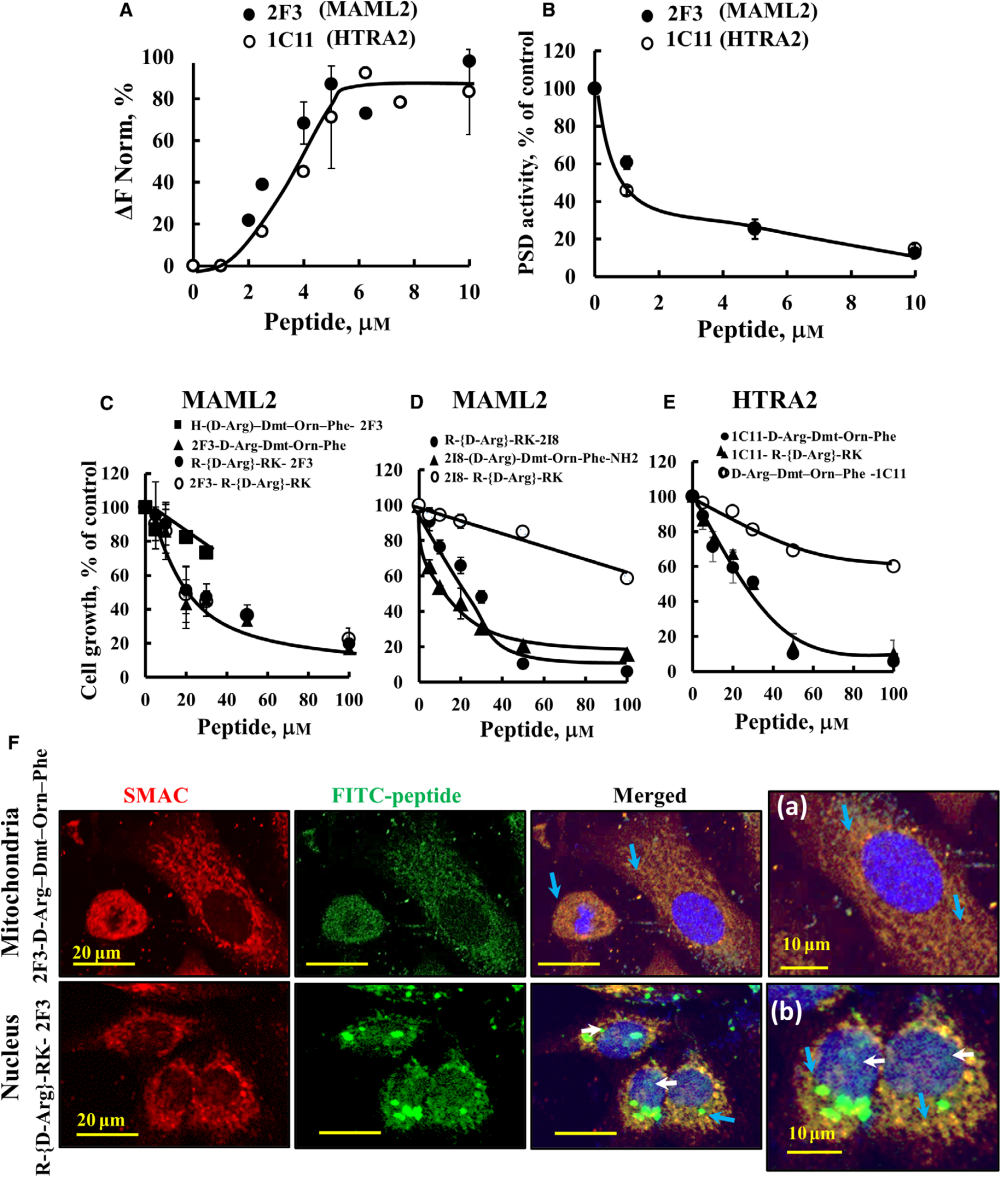
PSD‐interacting peptides bind to PSD, inhibiting its activity, and when cell‐penetrating and mitochondria‐ or nucleus‐targeted, inhibit cell proliferation. (A) Fluorescently‐labelled purified PSD (0.25 µm) was incubated with 2F3 (●) or 1C11 (○) peptide (1–10 µm) for 30 min at 37 °C; then, MST was used and revealed *K*_d_ of 3.0 µm for both peptides (*n* = 3). (B) Inhibition of PSD activity by the 2F3 (●) or 1C11 (○) peptide. PSD activity was measured using the DSB‐3 method. (C‐E) Cell proliferation inhibition following incubation of A549 cells with the indicated mitochondria‐ or nucleus‐targeted peptides for 24 h in a serum‐free medium, and cell proliferation was assayed using the SRB method. Results are the means ± SEM (*n* = 3). (F) A549 cells were incubated for 90 min with 5 µm of the mitochondria‐ or nucleus‐targeted FITC‐labelled peptides, immunostained with anti‐SMAC antibodies and with DAPI and visualized by confocal microscope for subcellular localization. (a) and (b) present higher magnification, white arrows point to possible nuclear localization of the peptide, and blue arrows to its colocalization with mitochondria marker (*n* = 3). Scale bars represent 20, 10 µm as indicated.

Purified PSD interacted with both synthetic peptides with a similar binding affinity (*K*_d_ of 3 µm), as monitored using MST (Fig. 7A) and inhibition of PSD activity with IC_50_ of 1 µm (Fig. 7B), suggesting specific interactions.

### 3.7 Cell‐penetrating PSD‐interacting peptides targeted to mitochondria or the nucleus inhibit cell proliferation

The peptides, 2F3 and IC11, derived from MAML2 and HTRA2, respectively, and 2I8 peptide derived from MAML2, but identified by PSD inhibiting SMAC binding to it, were designed as cell‐penetrating peptides (CPPs) targeted to mitochondria or nucleus and tested cell proliferation (Fig. 7C‐E, Table S4).

To target the peptide to the nucleus (nuCPP), the peptides were fused to the tetrapeptide RrRK (r = D‐arginine), shown to primarily target the nucleus of HeLa cells [34]. To target mitochondria, the peptides were fused to a mitochondria‐targeting sequence (mtCPP‐1) composed of D‐Arg–Dmt–Orn–Phe–NH_2_, where Dmt is 2,6‐dimethyl‐l‐tyrosine. mtCPP‐1 displayed several characteristics important for mitochondrial targeting: positive charge, lipophilicity and alternating aromatic and basic residues. mtCPP‐1 showed no toxicity in the different cell lines, and did not induce apoptosis [35].

The mtCPP‐2F3 peptide, derived from the nuclear protein MAML2, inhibited cell proliferation when the mitochondria‐targeting sequence was in the C terminus (IC_50,_ 19.6 µm), but no inhibition (IC_50,_ > 100 µm) was obtained when mtCPP was localized at the N terminus of the peptide (Fig. 7C, Table S4). In contrast, nuCPP added to the N‐ or the C terminus of 2F3 peptide resulted in similar cell proliferation inhibition (IC_50,_ 19.6 µm). Another peptide, 2I8, derived from MAML2, inhibited cell proliferation when the mitochondria‐ or nucleus‐targeting sequence was fused at the C terminus, with IC_50_ of 19.7 and 30 µm for the nucleus‐ and mitochondria‐targeting, respectively, but no significant inhibition was obtained when the mitochondria‐ or nucleus‐targeting sequence was at the N terminus (IC_50,_ > 100 and 67 µm, respectively) (Fig. 7D, Table S4).

Similar results were obtained with CPP‐IC11, inhibiting cell proliferation (IC_50,_ 30 µm) when the targeting sequence to mitochondria or the nucleus was localized to the C terminus, but not when fused to the N terminus of the peptide (Fig. 7E, Table S4).

Interestingly, peptide 1E14, derived from BIRC2 (mainly located to the cytosol) and identified by PSD‐preventing SMAC interaction, had no effect on cell proliferation when targeted to the mitochondria or the nucleus (Table S4).

Selected peptides were tested for their effect on noncancerous HaCaT cells showing no significant inhibition on cell proliferation (Fig. S6).

To demonstrate that the peptides reach the mitochondria or the nucleus, they were FITC‐labelled (Figs 7F and S7). Confocal images clearly show that the peptides were localized to the mitochondria or the nucleus, according to their targeting sequence. However, the nuclear‐targeted peptides also reached the mitochondria, most probably due to their high positive charge. Its presence in the nucleus can be seen when comparing the nucleus staining (Fig. 7E: a,b). Thus, peptides interacting with PSD inhibited cell proliferation when targeted to the mitochondria and/or nucleus.

### 3.8 PSD and SMAC are overexpressed in patient‐derived tumours and are localized to the nucleus

**Fig. 8:**
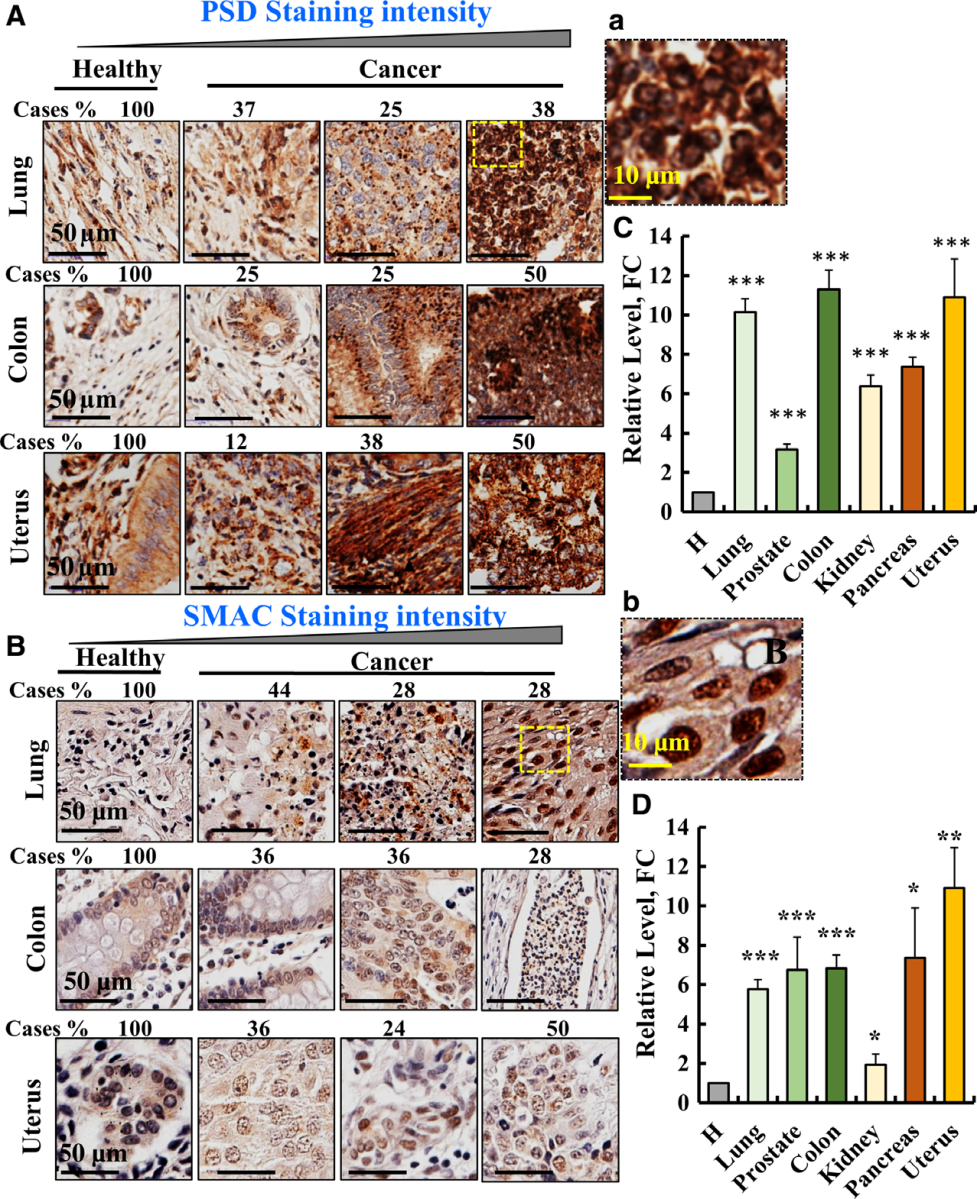
PSD and SMAC expression in tumours and presence in the nucleus. Representative IHC staining of PSD (A,C) and SMAC (B,D) in sections derived from the indicated healthy (*n* = 10) and cancer (*n* = 20) tissues in tissue microarray slides (US Biomax). The percentages of patient samples stained at the intensities presented in the scale are at the top of the figure, and the quantitative analysis is also shown (C,D). Image enlargements are given to show protein nuclear localization (Aa, Bb). Data shown are representative of three independent experiments. Results represent the means ± SEM, *P < 0.05, **P < 0.01, ***P < 0.001. Scale bars represent 50 or 10 µm as indicated.

Our results demonstrate that both PSD and SMAC were highly expressed in cancerous relative to noncancerous cell lines (Fig. 5D,E). Thus, we analysed the expression of SMAC and PSD in patient‐derived samples from different cancer types (Fig. 8). Both proteins were overexpressed (3‐ to 12‐fold) in various tumours including lung, colon, uterus, kidney, pancreas and prostate cancers, relative to their levels in healthy tissues (Fig. 8).

Interestingly, in lung cancer, both SMAC [21] and PSD were also found in the nucleus (Fig. 8A: a Fig. 8B: b). This agrees with PSD interacting with peptides derived from nuclear proteins such as MAML2, BIRC2, HTRA2, NR4A1, MTFR1 and ARNT (Tables 1 and S4).

To evaluate the presence of PSD in the nucleus, we selected H1563/CRL‐5875 (NCI‐H‐1563) cells showing the highest expression level of both SMAC and PSD (Fig. 5) (see https://www.oncomine.org). Applying nuclear fractionation, showing that both at the protein level and activity, PSD is found in the nuclear fraction (Fig. S8A,B), and thus, it may produce PE there as proposed previously [36].

We show that SMAC is present in tumours derived from patients (Fig. 1, 8). However, by nuclear fractionation in the cell line used (NCI‐H‐1563), SMAC was not clearly found in the nuclear fraction (Fig. S8A).

## 4 Discussion

Previously [21], we demonstrated, for the first time, that SMAC/Diablo depletion in cancer cells using specific si‐RNA led to multiple effects, including reduced cell proliferation and tumour growth, decreased phospholipid levels, and induced cell differentiation. These findings led us to propose that SMAC/Diablo possesses nonapoptotic functions associated with regulation of phospholipid synthesis [21].

Here, using CRISPR/Cas9 SMAC/Diablo depletion, we demonstrated inhibition of cancer cells proliferation, but not of cells considered as immortalized but noncancerous, and an increase in mitochondrial PE and decreased levels of other PLs. We discovered that SMAC regulates the levels of PE via direct interaction with mitochondria PSD, inhibiting its activity and identified PSD‐interacting sites with SMAC and three nuclear proteins. Peptides representing these sequences directly interact with and inhibit PSD activity. These interacting sequences were used to develop, for the first time, cytosol‐, mitochondria‐ or nucleus‐targeted peptides that inhibit PSD activity and cell proliferation.

**Fig. 9:**
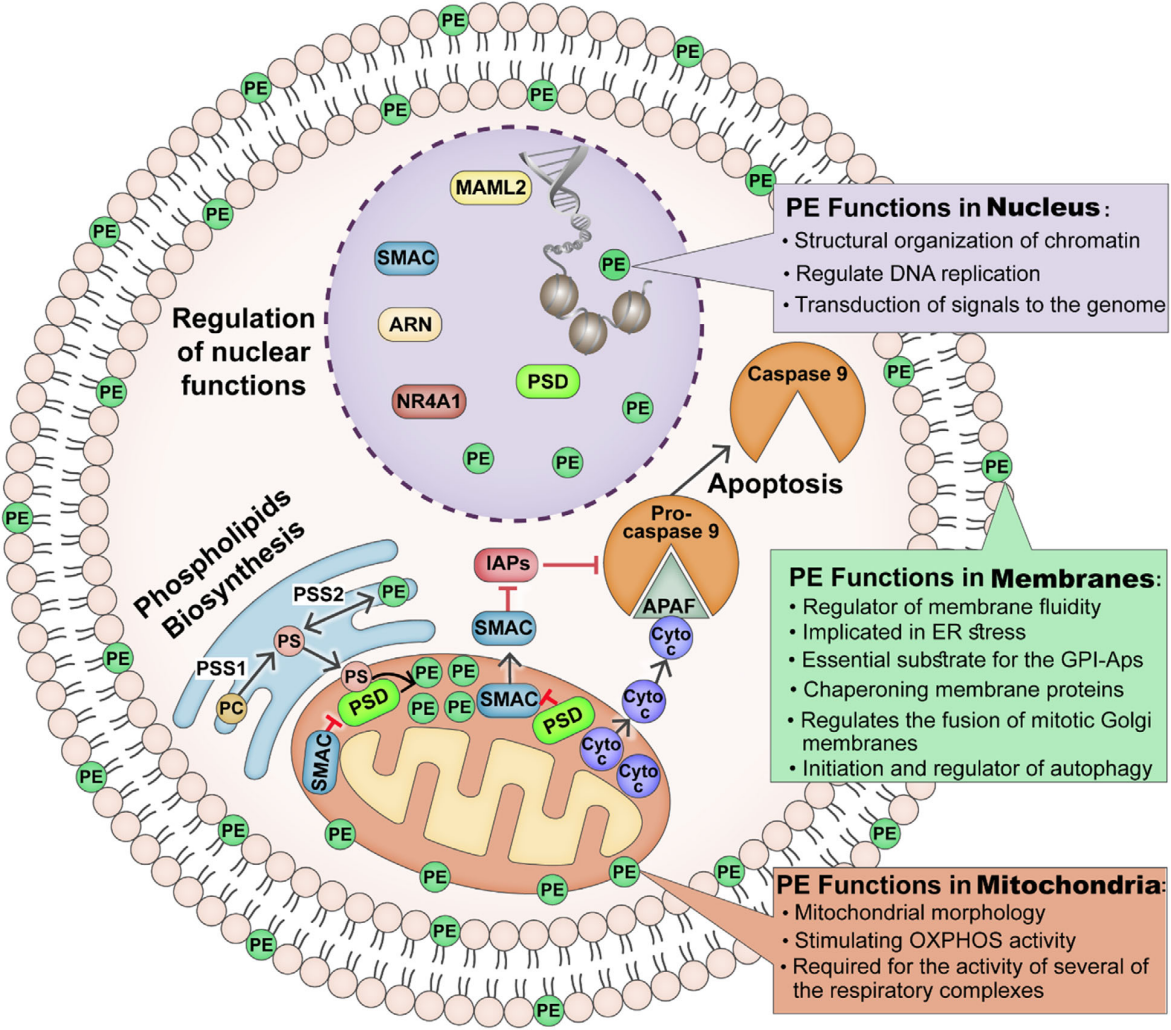
Proposed mode of PSD activity regulation of and by SMAC, affecting cellular PE levels and PE multifunction. A cell with ER, mitochondria and nucleus presenting SMAC, PSD and PE activities. Phospholipid biosynthesis: Mitochondria with proposed SMAC regulation of PSD activity, thereby regulating PE synthesis at ER‐mitochondria contact site (MAM). PS is produced in the ER from PC and PE, and PS then is transferred to the mitochondria, where it is converted by PSD into PE, and is depleted in other cell compartments including membranes and the nucleus. SMAC regulation of mitochondria‐mediated apoptosis and possible inhibition of SMAC release by PSD are proposed. PSD, SMAC and their identified interacting proteins in the nucleus, controlling PE production in nucleus. The PE multifunctions in the mitochondria, cell membrane, and nucleus are presented.

PE is not only a membrane component, but also is considered a signalling molecule that modulates structural organization of chromatin, nucleic acid synthesis and DNA replication [36, 37] (Fig. 9). Regulating PE levels, as demonstrated here, via SMAC and peptides modulating PSD activity resulted in changes in phospholipid levels in the cell, ER and nucleus, with PE being depleted in these membranes, but accumulating in the mitochondria.

Finally, while approaches for cancer treatment targeting metabolism, signal transduction and mutated proteins have long been addressed, this is the first time that phospholipid synthesis, found essential for cancer growth and tumour progression, is presented as an anticancer target.

Here, we show for the first time SMAC modulation of mitochondrial and nuclear PSD activity. In mammalian cells, PE can be synthesized by two major pathways: the Kennedy pathway, where the final step takes place on ER membranes by choline/ethanolamine phosphotransferase 1 (CEPT1) [38], and by decarboxylation of PS by the mitochondrial PSD [11]. PS is produced in the ER from PC and PE by PSS1 and PSS2, respectively, and then transferred to the mitochondria, where it is converted into PE by PSD (Figs 4C and 9). This key protein in PE synthesis is localized as SMAC in the IMS (Fig. 5A,B). SMAC directly interacts with and modulates PSD activity, as demonstrated by MST and PLA methods, and SMAC‐inhibiting PSD activity. Moreover, SMAC deletion both in tumours and in CRISPR/Cas9 SMAC‐KO cancer cells, resulted in about twofold decrease in PLs and PC levels, while PE levels were increased twofold in the mitochondria. These, together with increased PSD activity in the absence of SMAC, suggests that in the absence of SMAC, PSD is activated.

The increase in ER–mitochondria contact sites (MAM), the major site of PE synthesis, upon SMAC depletion (Fig. 3), suggests the formation of more sites for PS transport from the ER to the mitochondria and its conversion to PE by PSD. This, together with the high increase in mitochondria PE levels, suggests that in the absence of SMAC, the flux of PS from the ER to the mitochondria is increased. This, in turn, would result in depletion of PC, PE serving as PS precursors in the ER, as reflected in their decreased levels in the tumours and in SMAC‐KO cells.

The differences between cancer and nontumourigenic cells with respect to the differential effects of SMAC‐KO in HEK293T and A549 can be related to the presence of higher levels of PE, PSD in tumours from different cancer types (Fig. 8), and SMAC in cancer cell [21] (Fig. 5E‐G). In this respect, PE levels are elevated in several cancers [10] and PE is asymmetrically distributed in the inner leaflet of the plasma membrane of noncancer mammalian cells, but not in cancer cells which have lost their capacity to maintain PE asymmetry [7]. Additionally, IMS serine protease LACTB, leading to PSD degradation, changes mitochondrial lipid metabolism in certain cancer cells, but not in nontumourigenic differentiated cells [39]. Furthermore, LACTB‐mediated tumour suppression by increasing mitochondrial lipid metabolism [40]. Finally, acute myeloid leukaemia (AML) cells lacking PSD failed to form tumours [41].

Another important finding is that PE not only a membrane component, but also a signalling molecule. PE is the only phospholipid synthesized in mitochondria in addition to the ER [7]. PE has remarkable functions and has roles in autophagy [42, 43], ferroptosis, Parkinson's disease and cancer [44]. Overall, PE’s numerous functions include, but are not limited to (Fig. 9):

### 4.1 Membranes

PE is a regulator of membrane fluidity [45], with the ratio of PC to PE influencing membrane integrity and diseases like steatohepatitis [46]. The IMM is enriched in PE compared to other membranes, and a decrease in the mitochondrial content of PE profoundly alters mitochondrial morphology in mammalian cells [16]. PE is a lipid chaperone that assists in the folding of certain membrane proteins [47]. Finally, PE is an essential substrate for the synthesis of glycosylphosphatidylinositols (GPI‐Aps) acting as membrane anchors of many eukaryotic cell surface proteins [48].

### 4.2 Cell functions

PE plays an important role in several crucial cell functions such as cytokinesis [49], and a lack of PE causes cell cycle arrest [50]. PE also regulates the fusion of mitotic Golgi membranes [51] and is the source of ethanolamine that is covalently bound to the eukaryotic elongation factor eEF1A [52]. PE is required for the activity of several respiratory complexes supporting OXPHOS [9, 53]. It plays a key role in autophagy via covalently binding to cytosolic LC3 to form membrane‐bound LC3‐II, which is recruited to autophagosomal membranes [42, 43]. Moreover, mTOR inhibitors enhance both phospholipid remodelling and autophagy that depends on PSD activity and mitochondrial PE [54]. These PE functions may explain why SMAC depletion in tumours leads to exhaustion of cell PE while accumulating in the mitochondria, with pronounced decreased vesicle formation, altering nuclear morphology increased MAM, as well as inhibiting cell proliferation. Finally, PE plays a major role in cancer cell survival during metabolic stress conditions [55], implicated in ER stress relating to diabetes and neurodegeneration [56]. Furthermore, PE is associated with diseases [44], as its levels in substantia nigra pars compacta of Parkinson’s disease patients is significantly lower compared with healthy subjects [44].

### 4.3 Nuclear functions

The presence of chromatin‐associated phospholipid and the role of intranuclear lipids have been reported in several studies (reviewed in Ref. [57]). Lipid metabolism in nuclei is very active and is involved in signal transduction into the nucleus in response to agonists acting at the plasma membrane level [58]. The presence of PL in chromatin and the nuclear matrix, and the roles of nuclear PL in the structural organization of chromatin and nucleic acid synthesis have been demonstrated [36]. PL affects DNA [59] and RNA [60] metabolism, and PL content in the nuclei depends on the phase of DNA replication [37]. Here, we demonstrated the presence of PE, SMAC and PSD, and their interacting/regulatory proteins, BIRC2, TRAF‐2 and ARNT (Table 1) in the nucleus, thereby regulating PE levels as a signalling molecule the nucleus. As intranuclear PLs regulate DNA replication [37], upon SMAC depletion, changes in the expression of genes associated with the cell membrane, exosomes and ER‐ and Golgi‐related proteins and capillary organization [21] may result from the decrease in phospholipid levels in the cell and, thus, in the nucleus.

The nuclear localization of PSD and SMAC found here suggests a possible function of these proteins in the nucleus. Intranuclear lipids were found to function both in health and disease conditions [61] and numerous enzymes that synthesize and catabolize lipids were discovered within the nucleus [62]. Previously, we showed SMAC nuclear localization [21] and that its presence in the nucleus is a signature for squamous cell carcinoma (SCC), subtypes of non‐small‐cell lung cancer, as revealed by IHC and nuclear fractionation [63]. In contrast, PSD was not previously shown to be located in the nucleus, yet PE is proposed to be present there and its levels were increased in active chromatin [57].

In different tumours from patients (Fig. 8), both SMAC and PSD are overexpressed and localized also in the nucleus where PSD produces PE that regulates several nuclear properties and activities [36, 37, 59, 60]. It is not clear what causes the translocation of PSD and SMAC into the nucleus, and their retention there most probably is mediated by binding to nucleus‐located proteins. Indeed, we showed that PSD interacts with proteins also found in the nucleus, MAML2, HTRA2 and BIRC2. Thus, complex and dynamic interactions between PSD, SMAC and these proteins may take place in the nucleus, resulting in PE level modulation. Further studies are necessary to understand the mechanisms regulating PSD trafficking into and out of the nucleus and PSD functions in the nucleus under physiological and pathophysiology conditions such as cancer and other diseases.

Lipid is capable of regulating gene transcription, DNA replication or repair, and DNA cleavage, thereby regulating cell proliferation, differentiation, apoptosis and other cell functions [64]. Indeed, PLs and the enzyme precursors required for their synthesis were found in the nucleus as PIP2 [65], polyphosphoinositide‐metabolizing enzymes, kinases, phosphatases, phospholipases [65, 66, 67] and PKC [68]. Several metabolism‐associated proteins were reported to be present in the nucleus including the mitochondrial pyruvate‐dehydrogenase complex, generating locally acetyl‐CoA to fuel histone acetylation [69] or lipid biosynthesis [70]. Similarly, nuclear ATP‐citrate lyase generating acetyl‐CoA facilitating histone acetylation [71], acetyl‐CoA synthetase‐2 [72], thereby epigenetically regulating gene expression.

Thus, nuclear localization of PSD, SMAC and some of their interacting proteins (BIRC2, MAML2, HTRA2), along with PE, point to the importance of PE production and its regulation of nuclear and cell functions (Fig. 9).

Our results point to PSD as target for cancer therapy, as demonstrated by the inhibition of its activity and cell proliferation by novel PSD‐interacting peptides. Rapidly growing tumour cells require not only an abundance of metabolites and nucleotides, but also PLs for membrane biogenesis and tumour development [4]. Limiting the availability of membrane lipids could be an efficient mechanism to inhibit dividing cells [73]. Our results provide compelling evidence that a mitochondrial phospholipid metabolism is linked to cell proliferation and tumour formation. Based on the differences that exist between the membranes of healthy cells and tumour cells [3, 4, 5, 6], targeting membrane lipids in cancer cells is a promising approach towards an anticancer drug. PE is an attractive target for anticancer therapy [30]. It has been proposed as a target for peptides and small molecules such as duramycin and cinnamycin [29], and the natural product, ophiobolin A (OPA) [74] which binds specifically to the PE molecules on the cancer cell membrane, subsequently leading t to cell lysis [30].

Here, we propose to target mitochondrial PSD, the PE‐producing enzyme. In spite of extensive efforts, no inhibitor targeting mammalian PSD yet exists. 4‐quinolinamine was identified as a new antimalarial drug inhibiting membrane biogenesis in this parasite via inhibition of PSD activity [75]. As PSD resides in the IMS and nucleus, its targeting drugs must be targeted to both the mitochondria and nucleus. Here, we demonstrated that mitochondria‐ and nucleus‐targeted peptides derived from PSD‐interacting partners, HTRA2, BIRC2 and MAML2, interact with PSD, inhibiting its activity and cell proliferation. These peptides are the first inhibitors of mammalian PSD to be described.

## 5 Conclusions

Phospholipid synthesis is essential for cancer growth and development [3, 4, 5, 6]. The mitochondria pro‐apoptotic protein SMAC/Diablo overexpressed in cancer possesses a new nonapoptotic function in regulating phospholipid synthesis. SMAC/Diablo directly interacts with and inhibits the activity of the mitochondrial PSD, catalysing the synthesis of PE. SMAC/Diablo downregulation leads to PSD overproducing mitochondrial PE, and whole‐cell depletion of PLs leading to inhibition of cancer cell proliferation. Peptides inhibiting PSD activity were developed and when targeted to the mitochondria or the nucleus, inhibit cancer cell proliferation, the first reported PSD inhibitors in mammals.

The study highlights the function of PE not only as a membrane component, but also a signalling molecule and the nucleus as a metabolically active organelle with PSD‐producing PE capable of regulating nucleus function. Thus, PSD can be considered as a new target for cancer therapy.

## Conflict of interest

The authors declare no conflict of interest.

## Author contributions

SKP, AP, and AS‐K performed the experiments and analysed the data; UB provided the PE analysis method and reagent; RZ involved in structure modelling; VB‐S provided experimental oversight, interpreted the data, supervision and writing, reviewing and editing the manuscript.

### Peer Review

The peer review history for this article is available at https://publons.com/publon/10.1002/1878‐0261.12959.

## Supporting information


**Table␣S1**. Antibodies used in the study.
**Table␣S2**. Analysis of PL, PC, and PE levels in cell extract, mitochondria‐free, and mitochondria‐enriched fractions obtained from A549 cells expression or KO for SMAC.
**Table␣S3**. Proteins interacting with SMAC/Diablo as identified using a peptide array.
**Table␣S4**. Effects of peptide identified as interacting with PSD or their interaction with SMAC/Diablo is prevented in the presence of PSD on A549 lung cancer cell growth.
**Fig.␣S1**. Validation of PLA specificity, using SMAC and ATP synthase 5A.
**Fig.␣S2**. SMAC cell depletion resulted in increased cell and nucleus sizes.
**Fig.␣S3**. Confocal fluorescence imaging of PE as stained with DSB‐3.
**Fig.␣S4**. Peptides sequences directly interact with PSD (A) or PSD prevented their interaction with SMAC (B).
**Fig.␣S5**. Peptides identified from direct interact with PSD or PSD prevented their interaction with SMAC labelled in the proteins they derived from.
**Fig.␣S6**. PSD‐interacting peptides do not inhibit cell growth of epithelial HaCaT cells.
**Fig.␣S7**. 2I8 peptide targeted to the nucleus or to the mitochondria reaches these compartments.
**Fig.␣S8**. PSD is present in cell nucleus.

## Data Availability

Data are contained within the article or Supporting information and the crude data are available on request.

## References

[1] Baenke F , Peck B , Miess H & Schulze A (2013) Hooked on fat: the role of lipid synthesis in cancer metabolism and tumour development. Dis Model Mech 6, 1353–1363. 24203995 10.1242/dmm.011338 PMC3820259

[2] Shevchenko A & Simons K (2010) Lipidomics: coming to grips with lipid diversity. Nat Rev Mol Cell Biol 11, 593–598. 20606693 10.1038/nrm2934

[3] Adibhatla RM , Hatcher JF & Dempsey RJ (2006) Lipids and lipidomics in brain injury and diseases. AAPS J 8, E314–E321. 16796382 10.1007/BF02854902 PMC3231558

[4] Dobrzynska I , Szachowicz‐Petelska B , Darewicz B & Figaszewski ZA (2015) Characterization of human bladder cell membrane during cancer transformation. J Membr Biol 248, 301–307. 25572835 10.1007/s00232-015-9770-4 PMC4381039

[5] Wenk MR (2005) The emerging field of lipidomics. Nat Rev Drug Discov 4, 594–610. 16052242 10.1038/nrd1776

[6] Bandu R , Mok HJ & Kim KP (2018) Phospholipids as cancer biomarkers: Mass spectrometry‐based analysis. Mass Spectrom Rev 37, 107–138. 27276657 10.1002/mas.21510

[7] Vance JE & Tasseva G (2013) Formation and function of phosphatidylserine and phosphatidylethanolamine in mammalian cells. Biochim Biophys Acta 1831, 543–554. 22960354 10.1016/j.bbalip.2012.08.016

[8] Calzada E , Onguka O & Claypool SM (2016) Phosphatidylethanolamine Metabolism in Health and Disease. Int Rev Cell Mol Biol 321, 29–88. 26811286 10.1016/bs.ircmb.2015.10.001 PMC4778737

[9] Tasseva G , Bai HD , Davidescu M , Haromy A , Michelakis E & Vance JE (2013) Phosphatidylethanolamine deficiency in Mammalian mitochondria impairs oxidative phosphorylation and alters mitochondrial morphology. J Biol Chem 288, 4158–4173. 23250747 10.1074/jbc.M112.434183 PMC3567666

[10] Podo F (1999) Tumour phospholipid metabolism. NMR Biomed 12, 413–439. 10654290 10.1002/(sici)1099-1492(199911)12:7<413::aid-nbm587>3.0.co;2-u

[11] Percy AK , Moore JF , Carson MA & Waechter CJ (1983) Characterization of brain phosphatidylserine decarboxylase: localization in the mitochondrial inner membrane. Arch Biochem Biophys 223, 484–494. 6859873 10.1016/0003-9861(83)90613-6

[12] Vance JE (2014) MAM (mitochondria‐associated membranes) in mammalian cells: lipids and beyond. Biochim Biophys Acta 1841, 595–609. 24316057 10.1016/j.bbalip.2013.11.014

[13] Rusinol AE , Cui Z , Chen MH & Vance JE (1994) A unique mitochondria‐associated membrane fraction from rat liver has a high capacity for lipid synthesis and contains pre‐Golgi secretory proteins including nascent lipoproteins. J Biol Chem 269, 27494–27502. 7961664

[14] Achleitner G , Gaigg B , Krasser A , Kainersdorfer E , Kohlwein SD , Perktold A , Zellnig G & Daum G (1999) Association between the endoplasmic reticulum and mitochondria of yeast facilitates interorganelle transport of phospholipids through membrane contact. Eur J Biochem 264, 545–553. 10491102 10.1046/j.1432-1327.1999.00658.x

[15] Di Bartolomeo F , Wagner A & Daum G (2017) Cell biology, physiology and enzymology of phosphatidylserine decarboxylase. Biochim Biophys Acta Mol Cell Biol Lipids 1862, 25–38. 27650064 10.1016/j.bbalip.2016.09.007

[16] Steenbergen R , Nanowski TS , Beigneux A , Kulinski A , Young SG & Vance JE (2005) Disruption of the phosphatidylserine decarboxylase gene in mice causes embryonic lethality and mitochondrial defects. J Biol Chem 280, 40032–40040. 16192276 10.1074/jbc.M506510200 PMC2888304

[17] Du C , Fang M , Li Y , Li L & Wang X (2000) Smac, a mitochondrial protein that promotes cytochrome c‐dependent caspase activation by eliminating IAP inhibition. Cell 102, 33–42. 10929711 10.1016/s0092-8674(00)00008-8

[18] Verhagen AM , Ekert PG , Pakusch M , Silke J , Connolly LM , Reid GE , Moritz RL , Simpson RJ & Vaux DL (2000) Identification of DIABLO, a mammalian protein that promotes apoptosis by binding to and antagonizing IAP proteins. Cell 102, 43–53. 10929712 10.1016/s0092-8674(00)00009-x

[19] Kempkensteffen C , Hinz S , Christoph F , Krause H , Magheli A , Schrader M , Schostak M , Miller K & Weikert S (2008) Expression levels of the mitochondrial IAP antagonists Smac/DIABLO and Omi/HtrA2 in clear‐cell renal cell carcinomas and their prognostic value. J Cancer Res Clin Oncol 134, 543–550. 17922292 10.1007/s00432-007-0317-7 PMC12161611

[20] Yoo NJ , Kim HS , Kim SY , Park WS , Park CH , Jeon H , Jung ES , Lee JY & Lee SH (2003) Immunohistochemical analysis of Smac/DIABLO expression in human carcinomas and sarcomas. APMIS 111, 382–388. 12752217 10.1034/j.1600-0463.2003.t01-1-1110202.x

[21] Paul A , Krelin Y , Arif T , Jeger R & Shoshan‐Barmatz V (2018) A new role for the mitochondrial pro‐apoptotic protein SMAC/Diablo in phospholipid synthesis associated with tumourigenesis. Mol Ther 26, 680–694. 29396267 10.1016/j.ymthe.2017.12.020 PMC5910671

[22] Folch J , Lees M & Sloane Stanley GH (1957) A simple method for the isolation and purification of total lipides from animal tissues. J Biol Chem 226, 497–509. 13428781

[23] Choi JY , Surovtseva YV , Van Sickle SM , Kumpf J , Bunz UHF , Ben Mamoun C & Voelker DR (2018) A novel fluorescence assay for measuring phosphatidylserine decarboxylase catalysis. J Biol Chem 293, 1493–1503. 29247006 10.1074/jbc.RA117.000525 PMC5798280

[24] Gustafsdottir SM , Schallmeiner E , Fredriksson S , Gullberg M , Söderberg O , Jarvius M , Jarvius J , Howell M & Landegren U (2005) Proximity ligation assays for sensitive and specific protein analyses. Anal Biochem 345, 2–9. 15950911 10.1016/j.ab.2005.01.018

[25] Wienken CJ , Baaske P , Rothbauer U , Braun D & Duhr S (2010) Protein‐binding assays in biological liquids using microscale thermophoresis. Nat Commun 1, 100. 20981028 10.1038/ncomms1093

[26] Soderberg O , Gullberg M , Jarvius M , Ridderstrale K , Leuchowius KJ , Jarvius J , Wester K , Hydbring P , Bahram F , Larsson LG *et␣al*. (2006) Direct observation of individual endogenous protein complexes in␣situ by proximity ligation. Nat Methods 3, 995–1000. 17072308 10.1038/nmeth947

[27] Arbiser JL , Karalis K , Viswanathan A , Koike C , Anand‐Apte B , Flynn E , Zetter B & Majzoub JA (1999) Corticotropin‐releasing hormone stimulates angiogenesis and epithelial tumour growth in the skin. J Invest Dermatol 113, 838–842. 10571742 10.1046/j.1523-1747.1999.00760.x

[28] Vance JE (2008) Phosphatidylserine and phosphatidylethanolamine in mammalian cells: two metabolically related aminophospholipids. J Lipid Res 49, 1377–1387. 18204094 10.1194/jlr.R700020-JLR200

[29] Zhao M (2011) Lantibiotics as probes for phosphatidylethanolamine. Amino Acids 41, 1071–1079. 21573677 10.1007/s00726-009-0386-9 PMC3866894

[30] Tan LT , Chan KG , Pusparajah P , Lee WL , Chuah LH , Khan TM , Lee LH & Goh BH (2017) Targeting membrane lipid a potential cancer cure? Front Pharmacol 8, 12. 28167913 10.3389/fphar.2017.00012 PMC5253362

[31] Pettersen EF , Goddard TD , Huang CC , Couch GS , Greenblatt DM , Meng EC & Ferrin TE (2004) UCSF Chimera–a visualization system for exploratory research and analysis. J Comput Chem 25, 1605–1612. 15264254 10.1002/jcc.20084

[32] Samuel T , Okada K , Hyer M , Welsh K , Zapata JM & Reed JC (2005) cIAP1 Localizes to the nuclear compartment and modulates the cell cycle. Cancer Res 65, 210–218. 15665297

[33] Seok SH , Lee W , Jiang L , Molugu K , Zheng A , Li Y , Park S , Bradfield CA & Xing Y (2017) Structural hierarchy controlling dimerization and target DNA recognition in the AHR transcriptional complex. Proc Natl Acad Sci USA 114, 5431–5436. 28396409 10.1073/pnas.1617035114 PMC5448172

[34] Puckett CA & Barton JK (2010) Targeting a ruthenium complex to the nucleus with short peptides. Bioorg Med Chem 18, 3564–3569. 20430627 10.1016/j.bmc.2010.03.081 PMC2873839

[35] Cerrato CP , Pirisinu M , Vlachos EN & Langel U (2015) Novel cell‐penetrating peptide targeting mitochondria. FASEB J 29, 4589–4599. 26195590 10.1096/fj.14-269225

[36] Alessenko AV & Burlakova EB (2002) Functional role of phospholipids in the nuclear events. Bioelectrochemistry 58, 13–21. 12401566 10.1016/s1567-5394(02)00135-4

[37] Maraldi NM , Santi S , Zini N , Ognibene A , Rizzoli R , Mazzotti G , Di Primio R , Bareggi R , Bertagnolo V , Pagliarini C *et␣al*. (1993) Decrease in nuclear phospholipids associated with DNA replication. J Cell Sci 104 (Pt 3), 853–859. 8314878 10.1242/jcs.104.3.853

[38] Vance JE (1990) Phospholipid synthesis in a membrane fraction associated with mitochondria. J Biol Chem 265, 7248–7256. 2332429

[39] Keckesova Z , Donaher JL , De Cock J , Freinkman E , Lingrell S , Bachovchin DA , Bierie B , Tischler V , Noske A , Okondo MC *et␣al*. (2017) LACTB is a tumour suppressor that modulates lipid metabolism and cell state. Nature 543, 681–686. 28329758 10.1038/nature21408 PMC6246920

[40] Cucchi D & Mauro C (2017) LACTB‐mediated tumour suppression by increased mitochondrial lipid metabolism. Cell Death Differ 24, 1137–1139. 28475178 10.1038/cdd.2017.60 PMC5520170

[41] Seneviratne AK , Xu M , Aristizabal Henao JJ , Fajardo VA , Hao Z , Voisin V , Xu GW , Hurren R , Kim S , MacLean N *et␣al*. (2019) The mitochondrial transacylase, tafazzin, regulates AML stemness by modulating intracellular levels of phospholipids. Cell Stem Cell 24, 1007. 31173706 10.1016/j.stem.2019.04.020

[42] Kabeya Y , Mizushima N , Yamamoto A , Oshitani‐Okamoto S , Ohsumi Y & Yoshimori T (2004) LC3, GABARAP and GATE16 localize to autophagosomal membrane depending on form‐II formation. J Cell Sci 117, 2805–2812. 15169837 10.1242/jcs.01131

[43] Rockenfeller P , Koska M , Pietrocola F , Minois N , Knittelfelder O , Sica V , Franz J , Carmona‐Gutierrez D , Kroemer G & Madeo F (2015) Phosphatidylethanolamine positively regulates autophagy and longevity. Cell Death Differ 22, 499–508. 25571976 10.1038/cdd.2014.219 PMC4326582

[44] Patel D & Witt SN (2017) Ethanolamine and phosphatidylethanolamine: partners in health and disease. Oxid Med Cell Longev 2017, 4829180. 28785375 10.1155/2017/4829180 PMC5529665

[45] Dawaliby R , Trubbia C , Delporte C , Noyon C , Ruysschaert JM , Van Antwerpen P & Govaerts C (2016) Phosphatidylethanolamine is a key regulator of membrane fluidity in eukaryotic cells. J Biol Chem 291, 3658–3667. 26663081 10.1074/jbc.M115.706523 PMC4751403

[46] Li Z , Agellon LB , Allen TM , Umeda M , Jewell L , Mason A & Vance DE (2006) The ratio of phosphatidylcholine to phosphatidylethanolamine influences membrane integrity and steatohepatitis. Cell Metab 3, 321–331. 16679290 10.1016/j.cmet.2006.03.007

[47] Bogdanov M , Umeda M & Dowhan W (1999) Phospholipid‐assisted refolding of an integral membrane protein. Minimum structural features for phosphatidylethanolamine to act as a molecular chaperone. J Biol Chem 274, 12339–12345. 10212204 10.1074/jbc.274.18.12339

[48] Kinoshita T & Fujita M (2016) Biosynthesis of GPI‐anchored proteins: special emphasis on GPI lipid remodeling. J Lipid Res 57, 6–24. 26563290 10.1194/jlr.R063313 PMC4689344

[49] Emoto K & Umeda M (2000) An essential role for a membrane lipid in cytokinesis. Regulation of contractile ring disassembly by redistribution of phosphatidylethanolamine. J Cell Biol 149, 1215–1224. 10851019 10.1083/jcb.149.6.1215 PMC2175113

[50] Signorell A , Gluenz E , Rettig J , Schneider A , Shaw MK , Gull K & Butikofer P (2009) Perturbation of phosphatidylethanolamine synthesis affects mitochondrial morphology and cell‐cycle progression in procyclic‐form *Trypanosoma brucei* . Mol Microbiol 72, 1068–1079. 19400804 10.1111/j.1365-2958.2009.06713.x

[51] Pecheur EI , Martin I , Maier O , Bakowsky U , Ruysschaert JM & Hoekstra D (2002) Phospholipid species act as modulators in p97/p47‐mediated fusion of Golgi membranes. Biochemistry 41, 9813–9823. 12146947 10.1021/bi0259195

[52] Signorell A , Jelk J , Rauch M & Butikofer P (2008) Phosphatidylethanolamine is the precursor of the ethanolamine phosphoglycerol moiety bound to eukaryotic elongation factor 1A. J Biol Chem 283, 20320–20329. 18499667 10.1074/jbc.M802430200

[53] Shinzawa‐Itoh K , Aoyama H , Muramoto K , Terada H , Kurauchi T , Tadehara Y , Yamasaki A , Sugimura T , Kurono S , Tsujimoto K *et␣al*. (2007) Structures and physiological roles of 13 integral lipids of bovine heart cytochrome c oxidase. EMBO J 26, 1713–1725. 17332748 10.1038/sj.emboj.7601618 PMC1829383

[54] Thomas HE , Zhang Y , Stefely JA , Veiga SR , Thomas G , Kozma SC & Mercer CA (2018) Mitochondrial complex I activity is required for maximal autophagy. Cell Rep 24, 2404–2417, e2408. 30157433 10.1016/j.celrep.2018.07.101 PMC6298213

[55] Zhu L & Bakovic M (2012) Breast cancer cells adapt to metabolic stress by increasing ethanolamine phospholipid synthesis and CTP:ethanolaminephosphate cytidylyltransferase‐Pcyt2 activity. Biochem Cell Biol 90, 188–199. 22339418 10.1139/o11-081

[56] Fu S , Yang L , Li P , Hofmann O , Dicker L , Hide W , Lin X , Watkins SM , Ivanov AR & Hotamisligil GS (2011) Aberrant lipid metabolism disrupts calcium homeostasis causing liver endoplasmic reticulum stress in obesity. Nature 473, 528–531. 21532591 10.1038/nature09968 PMC3102791

[57] Albi E & Viola Magni MP (2004) The role of intranuclear lipids. Biol Cell 96, 657–667. 15519699 10.1016/j.biolcel.2004.05.004

[58] D'Santos CS , Clarke JH & Divecha N (1998) Phospholipid signalling in the nucleus. Een DAG uit het leven van de inositide signalering in de nucleus. Biochim Biophys Acta 1436, 201–232. 9838115 10.1016/s0005-2760(98)00146-5

[59] Shoji‐Kawaguchi M , Izuta S , Tamiya‐Koizumi K , Suzuki M & Yoshida S (1995) Selective inhibition of DNA polymerase epsilon by phosphatidylinositol. J Biochem 117, 1095–1099. 8586625 10.1093/oxfordjournals.jbchem.a124812

[60] Albi E , Micheli M & Viola Magni MP (1996) Phospholipids and nuclear RNA. Cell Biol Int 20, 407–412. 8858825 10.1006/cbir.1996.0051

[61] Albi E (2011) Role of intranuclear lipids in health and disease. Clin Lipidol 6, 59–69.

[62] Ledeen L & Wu G (2006) Nuclear lipids and their metabolic and signaling properties. In Handbook of Neurochemistry and Molecular Neurobiology. 3 edn, pp. 173–198. Springer, Boston, MA.

[63] Shoshan‐Barmatz V , Bishitz Y , Paul A , Krelin Y , Nakdimon I , Peled N , Lavon A , Rudoy‐Zilberman E & Refaely Y (2017) A molecular signature of lung cancer: potential biomarkers for adenocarcinoma and squamous cell carcinoma. Oncotarget 8, 105492–105509. 29285267 10.18632/oncotarget.22298 PMC5739654

[64] Faenza I , Fiume R , Piazzi M , Colantoni A & Cocco L (2013) Nuclear inositide specific phospholipase C signalling ‐ interactions and activity. FEBS J 280, 6311–6321. 23890371 10.1111/febs.12450

[65] Keune W , Bultsma Y , Sommer L , Jones D & Divecha N (2011) Phosphoinositide signalling in the nucleus. Adv Enzyme Regul 51, 91–99. 21035491 10.1016/j.advenzreg.2010.09.009

[66] Martelli AM , Ognibene A , Buontempo F , Fini M , Bressanin D , Goto K , McCubrey JA , Cocco L & Evangelisti C (2011) Nuclear phosphoinositides and their roles in cell biology and disease. Crit Rev Biochem Mol Biol 46, 436–457. 21913876 10.3109/10409238.2011.609530

[67] Shah ZH , Jones DR , Sommer L , Foulger R , Bultsma Y , D'Santos C & Divecha N (2013) Nuclear phosphoinositides and their impact on nuclear functions. FEBS J 280, 6295–6310. 24112514 10.1111/febs.12543

[68] Nishizuka Y (1995) Protein kinase C and lipid signaling for sustained cellular responses. FASEB J 9, 484–496. 7737456

[69] Sutendra G , Kinnaird A , Dromparis P , Paulin R , Stenson TH , Haromy A , Hashimoto K , Zhang N , Flaim E & Michelakis ED (2014) A nuclear pyruvate dehydrogenase complex is important for the generation of acetyl‐CoA and histone acetylation. Cell 158, 84–97. 24995980 10.1016/j.cell.2014.04.046

[70] Chen J , Guccini I , Di Mitri D , Brina D , Revandkar A , Sarti M , Pasquini E , Alajati A , Pinton S , Losa M *et␣al*. (2018) Compartmentalized activities of the pyruvate dehydrogenase complex sustain lipogenesis in prostate cancer. Nat Genet 50, 219–228. 29335542 10.1038/s41588-017-0026-3 PMC5810912

[71] Sivanand S , Rhoades S , Jiang Q , Lee JV , Benci J , Zhang J , Yuan S , Viney I , Zhao S , Carrer A *et␣al*. (2017) Nuclear Acetyl‐CoA production by ACLY promotes homologous recombination. Mol Cell 67, 252–265.e256. 28689661 10.1016/j.molcel.2017.06.008 PMC5580398

[72] Bulusu V , Tumanov S , Michalopoulou E , van den Broek NJ , MacKay G , Nixon C , Dhayade S , Schug ZT , Vande Voorde J , Blyth K *et␣al*. (2017) Acetate recapturing by nuclear acetyl‐CoA synthetase 2 prevents loss of histone acetylation during oxygen and serum limitation. Cell Rep 18, 647–658. 28099844 10.1016/j.celrep.2016.12.055 PMC5276806

[73] van Meer G , Voelker DR & Feigenson GW (2008) Membrane lipids: where they are and how they behave. Nat Rev Mol Cell Biol 9, 112–124. 18216768 10.1038/nrm2330 PMC2642958

[74] Chidley C , Trauger SA , Birsoy K & O'Shea EK (2016) The anticancer natural product ophiobolin A induces cytotoxicity by covalent modification of phosphatidylethanolamine. Elife 5, e14601. 27403889 10.7554/eLife.14601 PMC4942256

[75] Choi JY , Kumar V , Pachikara N , Garg A , Lawres L , Toh JY , Voelker DR & Ben Mamoun C (2016) Characterization of Plasmodium phosphatidylserine decarboxylase expressed in yeast and application for inhibitor screening. Mol Microbiol 99, 999–1014. 26585333 10.1111/mmi.13280 PMC4898484

[76] Vande Walle L , Lamkanfi M & Vandenabeele P (2008) The mitochondrial serine protease HtrA2/Omi: an overview. Cell Death Differ 15, 453–460. 18174901 10.1038/sj.cdd.4402291

[77] Wu L , Sun T , Kobayashi K , Gao P & Griffin JD (2002) Identification of a family of mastermind‐like transcriptional coactivators for mammalian notch receptors. Mol Cell Biol 22, 7688–7700. 12370315 10.1128/MCB.22.21.7688-7700.2002 PMC135662

[78] Zhang L , Wang Q , Liu W , Liu F , Ji A & Li Y (2018) The orphan nuclear receptor 4A1: a potential new therapeutic target for metabolic diseases. J Diabetes Res 2018, 9363461. 30013988 10.1155/2018/9363461 PMC6022324

