# Supplementary material for: SMAC/Diablo controls proliferation of cancer cells by regulating phosphatidylethanolamine synthesis
Source: Mol Oncol. 2021 May 4;15(11):3037–61. doi: 10.1002/1878-0261.12959 (PMC8564633; doi:10.1002/1878-0261.12959)
Supplement: Supplementary file 1 — Table␣S1. Antibodies used in the study. Table␣S2. Analysis of PL, PC, and PE levels in cell extract, mitochondria‐free, and mitochondria‐enriched fractions obtained from A549 cells expression or KO for SMAC. Table␣S3. Proteins interacting with SMAC/Diablo as identified using a peptide array. Table␣S4. Effects of peptide identified as interacting with PSD or their interaction with SMAC/Diablo is prevented in the presence of PSD on A549 lung cancer cell growth. Fig.␣S1. Validation of PLA specificity, using SMAC and ATP synthase 5A. Fig.␣S2. SMAC cell depletion resulted in increased cell and nucleus sizes. Fig.␣S3. Confocal fluorescence imaging of PE as stained with DSB‐3. Fig.␣S4. Peptides sequences directly interact with PSD (A) or PSD prevented their interaction with SMAC (B). Fig.␣S5. Peptides identified from direct interact with PSD or PSD prevented their interaction with SMAC labelled in the proteins they derived from. Fig.␣S6. PSD‐interacting peptides do not inhibit cell growth of epithelial HaCaT cells. Fig.␣S7. 2I8 peptide targeted to the nucleus or to the mitochondria reaches these compartments. Fig.␣S8. PSD is present in cell nucleus. [file MOL2-15-3037-s001.pdf]

Supplementary Data

Table S1. Antibodies used in the study

Antibodies against the specific protein, source, catalogue number, and the dilutions used in immunohistochemistry (IHC), immunofluorescence (IF) and immunoblot (WB) are presented.

Antibody	Source and Cat. No.	Dilution	
		IHC/IF	WB
Mouse monoclonal anti-actin	Millipore, Billerica, MA, MAB1501	-	1:20000
Rabbit polyclonal anti-SMAC/Diablo	Abcam, ab8115	1:500	1:2000
Rabbit monoclonal anti-Ki-67	Thermo Scientific, NY RM9106-s1	1:100	-
Mouse monoclonal anti-ATPsyn5a	Abcam, Cambridge, UK, ab14748	1:300	1:1000
Rabbit polyclonal anti-citrate synthase	Abcam, Cambridge, UK ab96600	1:200	1:4000
Rabbit monoclonal anti-VDAC1	Abcam, Cambridge, UK, ab154856	1:500	1:5000
Mouse monoclonal anti-VDAC1	Abcam, Cambridge, UK, ab186321	1:750	1:5000
Mouse monoclonal anti-GAPDH	Abcam, Cambridge, UK, ab9484	-	1:2000
Rabbit polyclonal anti-H4	Abcam, Cambridge, UK, ab10158	-	1:1000
Mouse monoclonal anti-PSD	Santa Cruz, TX, sc-390070	1:300	1:2000
Mouse monoclonal anti-PSD	Sigma, MO, HPA031090	1:500	1:3000
Rabbit polyclonal anti-IP3 receptor	Abcam, Cambridge, UK, ab5804	1:500	-
Anti-Rabbit IgG, HRP conjugate	Promega Corporation, WI, USA	1:1000	1:10000
Anti-Mouse IgG, HRP conjugate	Abcam, Cambridge, UK, ab98799	1:1000	1:10000
Anti-Mouse IgG, Alexa Fluor 488	Abcam, Cambridge, UK, ab150109	1:750	-
Anti-Mouse IgG, Alexa Fluor 555	Abcam, Cambridge, UK, ab 150110	1:750	-
Anti-Rabbit IgG, Alexa Fluor 555	Abcam, Cambridge, UK, ab150086	1:750	-
Anti-Rabbit IgG, Alexa Fluor 488	Thermo Fisher Scientific, USA, A-11008	1:750	-

Table S2. Analysis of PL, PC, and PE levels in cell extract, mitochondria-free, and mitochondria-enriched fractions obtained from A549 cells expression or KO for SMAC

Cells, their extracts and mitochondria-free, and mitochondria-enriched fractions were obtained as described in Materials and Methods. PL, PE and PC levels were analyzed as described in Materials and Methods. Calibration curves were used, using PC (Sigma Aldrich, St. Louis, MO) and PE (Biovision, CA) and inorganic phosphate (Pi) for total phospholipids. The results are the means ±SD (n=3).

No	Fraction	Phospholipid level, nmole/mg of protein		
		PL	PC	PE
1	A549 total cells extract	74.62±6.22	44.64±2.06	37.37±2.21
2	A549 mitochondria-free	29.80±1.12	15.36±0.03	24.52±1.0
3	A549 mitochondria	100.17±8.55	62.60±1.0	69.33±12.56
4	A549-KO total cells extract	39.55±2.88	21.35±0.55	77.12±13.6
5	A549-KO mitochondria-free	15.98±0.13	8.98±0.07	24.47±5.88
6	A549-KO-mitochondria	52.5±4.69	32.6±0.67	143.19±8.89

Table S3. Proteins interacted with SMAC/Diablo as identified using a peptide array

Eleven selected SMAC/Diablo interacting proteins based on BioGRID (https://thebiogrid.org/) were used in a peptide array (see Methods) and the nine proteins interacted with SMAC/Diablo was identified using the peptide array. For each protein, the Uniprot symbol, accession, other names, and intracellular localization are provided.

Protein (Uniport accession)	Proposed function	Location
**BIRC2 (cIAP1)- Baculoviral IAP repeat-containing protein 2 (Q13490)**	Regulates apoptosis via caspases activity and, mitogenic kinase signaling, and cell proliferation, cell invasion and metastasis. Modulates inflammatory signaling and immunity via E3 ubiquitin-protein ligase regulating both canonical and non-canonical NF-κ-B signaling, NEDD8 conjugation pathway, TLRs), Nod like receptors (NLRs) and RIG-I like receptors (RLRs).	Nucleus, Cytosol, plasma membrane
**BIRC5 (Survivin)- Baculoviral IAP repeat-containing protein 5 (Q15392)**	Promotes cell proliferation and preventing apoptosis. Involved in chromosome alignment and segregation during mitosis and cytokinesis	Nucleus, Cytosol, Cytoskeleton
**TRAF2- TNF receptor associated factor 2 (Q12933)**	Regulates activation of NF-κB and JNK, cell survival and apoptosis. Required for normal antibody isotype switching from IgM to IgG. Has E3 ubiquitin-protein ligase activity and regulates BIRC2, BIRC3, RIPK1 and TICAM1 protein levels by inhibiting their autoubiquitination	Cytosol
**UBE2K (HIP2)- ubiquitin conjugating enzyme E2 K (P61086)**	Involved in ubiquitination of NF-kB and mediates the selective degradation of short-lived and abnormal proteins, such as the ER-associated degradation (ERAD) of misfolded luminal proteins	Cytosol
**MAML2-mastermind like transcriptional coactivator 2 truncated poly Q (Q8IZL2)**	A transcriptional coactivator for NOTCH proteins. Has been shown to amplify NOTCH-induced transcription of HES1.	Nucleus
**ARNT (BHLHE1)-Aryl hydrocarbon receptor nuclear translocator (P27540)**	The ARNT forms a complex with ligand-bound aryl hydrocarbon receptor (AhR) and is required for receptor function. Identified as the beta subunit of a heterodimeric transcription factor, hypoxia-inducible factor 1 (HIF1) and functions as a transcriptional regulator of the adaptive response to hypoxia.	Nucleus
**NR4A1 (GFRP1)- Nuclear receptor subfamily 4 group A member 1 (P22736)**	Orphan nuclear receptor binds the NGFI-B response element, involved in the regulation of energy homeostasis, and attenuates AMPK activity in cytoplasm. Inhibits NF-kB activity	Nucleus, mitochondria
**CD40 (TNFSF5)- Tumor necrosis factor receptor superfamily member 5 (P25942)**	Receptor for TNFSF5/CD40LG. Transduces TRAF6- and MAP3K8-mediated signals that activate ERK in macrophages and B cells, leading to induction of immunoglobulin secretion.	Plasma membrane
**HTRA2- Serine protease HTRA2, mitochondrial (O43464)**	Serine protease works on a non-specific substrate β-casein. Promotes cell death either by an increase in caspase activity or direct binding to BIRC proteins and their inhibition	Mitochondrion intermembrane space, Nucleus

Table S4. Effects of peptide identified as interacting with PSD or their interaction with SMAC/Diablo is prevented in the presence of PSD on A549 lung cancer cell growth

A549 cells were seeded in 96-well plate (7000 cells/well). After 24 h cells were incubated without or with the indicated peptide concentration for 24 h in a serum-free medium. Then, cells were washed with PBS, and analyzed for cell growth using the SRB method. Results are the mean ± SD (n=3). Peptide sequence, its identification manner, based on interacting with PSD or their interaction with SMAC/Diablo is prevented in the presence of PSD are shown. In addition, the peptides are presented with the cell penetrating peptide targeting to the mitochondria or the nucleus. The results are presented as the concentration required for 50% inhibition (IC_{50}).

No	Peptide spot and sequence	Protein/Function	Peptide and Cellular Targeting	IC_{50} µM
1	**2F3** ALSTSSPIPSVPQSQ AQPQTGSGAS ***Interacting with PSD***	**MAML2** -mastermind like transcriptional coactivator 2 truncated poly Q, transcriptional coactivator for NOTCH proteins, promotes proliferative signaling during neurogenesis (Nucleus).	**2F3** -D-Arg–Dmt–Orn–Phe Mitochondria (A)	19.6±0.4
			H-(D-Arg)–Dmt–Orn–Phe- **2F3** Mitochondria	>100
			R-{D-Arg}-RK- **2F3** Nucleus	28.9±5.2
			**2F3-** R-{D-Arg}-RK Nucleus	29.6±0.6
2	**2I8** PNQSSRAFQGTDHS SDLAFDFLSQQ ***Identified by PSD preventing SMAC interaction with it***	**MAML2** mastermind like transcriptional coactivator 2 truncated poly Q, A transcriptional coactivator for NOTCH proteins, promotes proliferative signaling during neurogenesis (Nucleus).	**2I8**- (D-Arg)-Dmt-Orn-Phe-NH2 Mitochondria	30.2±3.6
			D-Arg–Dmt–Orn–Phe-**2I8** Mitochondria	66.8±4.7
			R-{D-Arg}-RK-**2I8** Nucleus	>100
			**2I8**- R-{D-Arg}-RK Nucleus	19.7±4.3
3	**1C11** ALLTSGTSDPRARV TYGTPSLWARL ***Interacting with PSD***	**HTRA2** -Serine peptidase 2, Serine protease promotes cell death either by direct binding to IAPs proteins leading to increased caspase activity or caspase-independent and serine protease activity-dependent mechanism. (Mitochondrion intermembrane space).	**1C11**-D-Arg–Dmt–Orn–Phe Mitochondria	30.3±5.5
			D-Arg–Dmt–Orn–Phe **-1C11** Mitochondria	89.3±5.49
			R-{D-Arg}-RK- **1C11** Nucleus	66.5±3.9
			**1C11-** R-{D-Arg}-RK Nucleus	30.0±3.8
4	**1E14** ELYRMSTYSTFPAG VPVSERSLARA ***Identified by PSD preventing SMAC interaction with it***	**BIRC2-** baculoviral IAP2 repeat. Acts as an E3 ubiquitin-protein ligase, regulator of NF-kappa-B signaling, apoptosis, cell proliferation, cell invasion and metastasis, modulates inflammatory signaling, (Cytosol, nucleus and plasma membrane).	D-Arg–Dmt–Orn–Phe-1E14 Mitochondria	>100
			1E14- D-Arg–Dmt–Orn–Phe Mitochondria	>100
			R-{D-Arg}-RK-1E14 Nucleus	>100

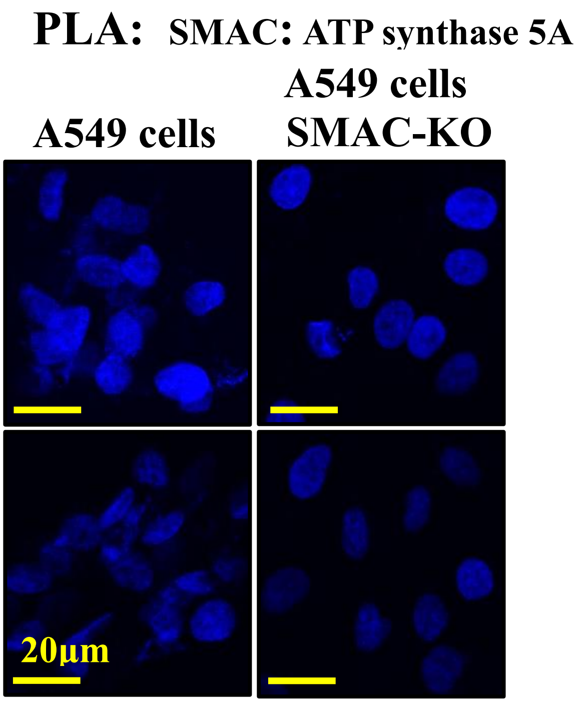

Figure S1. Validation of PLA specificity, using SMAC and ATP synthase 5A

Cells expressing SMAC were subjected to *in situ* PLA to test for the interaction between SMAC located at the intermembrane space and ATP synthase subunit 5A located at the inner mitochondrial membrane facing the matrix, using specific antibodies. PLA was carried out as in the Materials and Methods section. The ligation products are represented by red color (no product in this case), while nucleuses were DAPI-stained. Scale bar represents 20 µm.

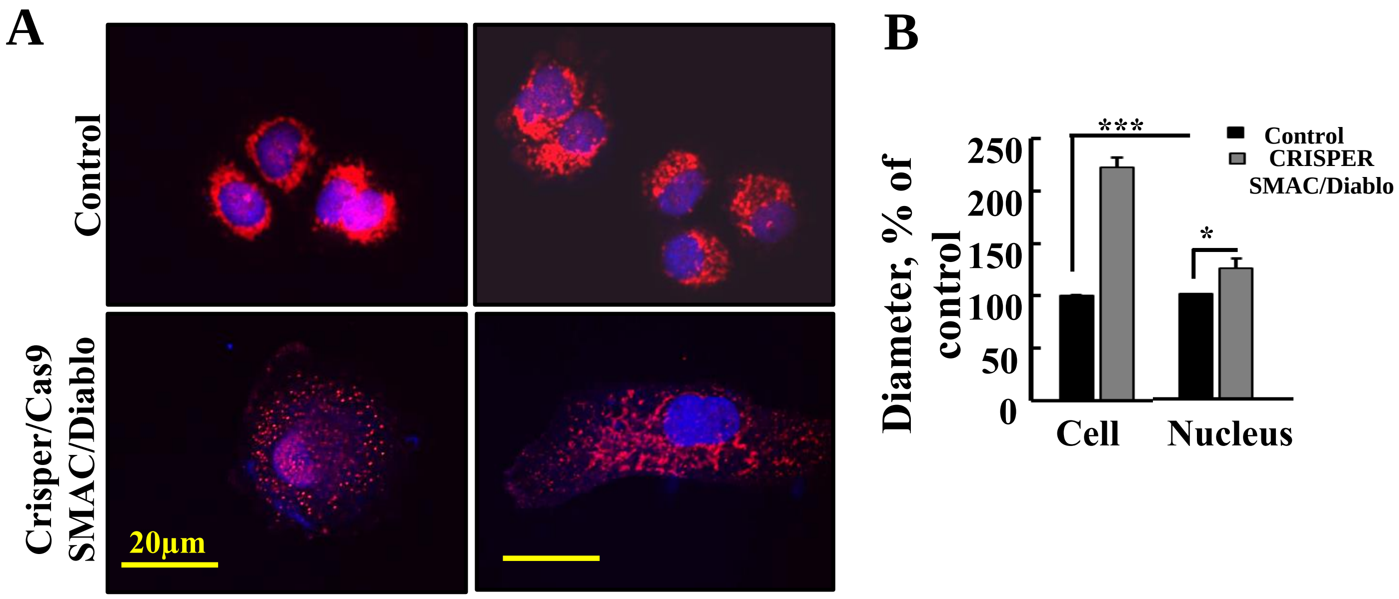

Figure S2. SMAC cell depletion resulted in increased cell and nucleus sizes

Control and CRISPR/Cas9-generated SMAC-deficient A549 cells were stained for SMAC using anti-SMAC antibodies and nucleus stained with DAPI (**A**). Cell and nucleus diameters of about 100 cells from control and CRISPR/Cas9-SMAC-deficient cells were measured (**B**). Results represent the means ± SEM (n=3), *P<0.05; ***P<0.001. Scale bar represents 20 µm.

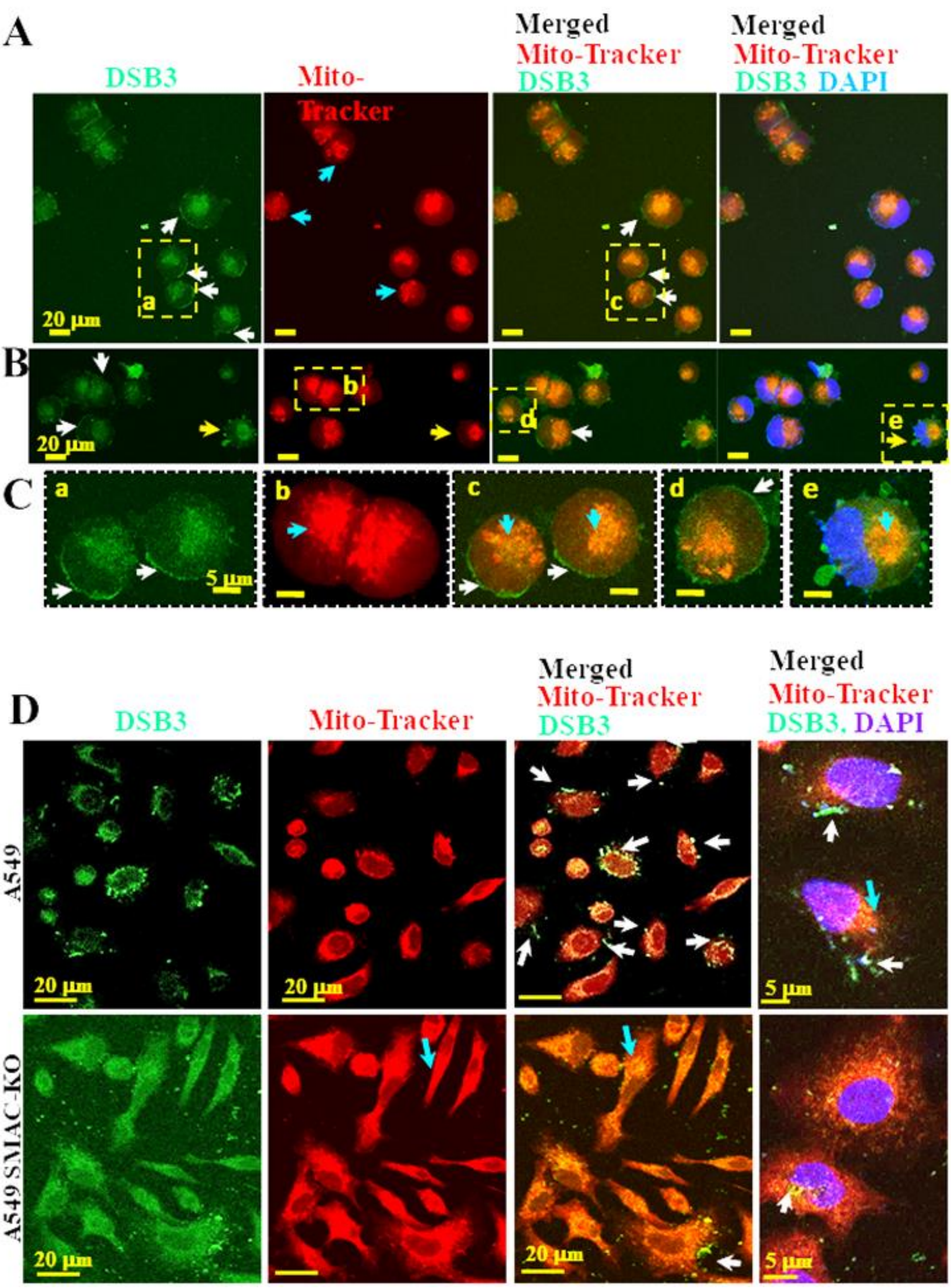

Figure S3. **Confocal fluorescence imaging of PE as stained with DSB-3**

A549 cells were stained with MitoTracker (250 nM) for 45 min followed by incubation with DSB-3 (10 µM) for 2h. Nuclei were stained with DAPI. (**A,B**) Representative images showing DSB-3-lebled PE at the plasma membrane and in the mitochondria. (**C**) Magnification of the squared sections in (**A,B**) to point to plasma membranes (white arrow, ***a, c, d***), mitochondria (blue arrows, ***b, c, e***), and apoptotic cell with blubbing membrane is shown in (***e***). (**D**) Control and SMAC-OK A549 cells stained with DSB-3, MitoTracker, and DAPI. White arrows point to PE and blue arrows to its co-localization with mitochondria marker, MitoTracker. The results are representative of three independent experiments. Scale bars represent 20 or 5 µm as indicated.

A. Peptides identified by blotting peptide-array with PSD

HTRA2

RPRLTPDLRALLTSGTSDPRARVTY
1C11– ALLTSGTSDPRARVTYGTPSLWRL
PRARVTYGTPSLWARLSVGVTEPRA

QLTAVTPDTRTREASENSGTRSRAW
1C17– RTREASENSGTRSRAWLAVALGAGG
GTRSRAWLAVALGAGGAVLLLLWGG

BIRC2

SRTNPYSYAMSTEEARFLTYHMWPL
1F5– MSTEEARFLTYHMWPLTFLSPSELA
TYHMWPLTFLSPSELARAGFYYIGP

MTFR1

MPNMLEILKEMNSVKLRSVKRSEQD
2B4– EMNSVKLRSVKRSEQDVKPKPVDAT
VKRSEQDVKPKPVDATDPAALIAEA

MAML2

GPAFSMANSALSTSSPIPSVPQSQA
2F3– ALSTSSPIPSVPQSQAQPQTGSGAS
2F4– SVPQSQAQPQTGSGASRALPSWQEV
QTGSGASRALPSWQEVSHAQQLKQI

ARNT

PGRDGLASYNHSQVVQPVTTTGPEH
2K24– NHSQVVQPVTTTGPEHSKPLEKSDG
TTTGPEHSKPLEKSDGLFAQDRDPR

NR4A1

QPGSFCWALKADGIMWLAKACWSIQ
1L9– KADGIMWLAKACWSIQSEMPCIQAQ
KACWSIQSEMPCIQAQYGTPAPSPG

GDNASCQHYGVRTCEGCKGFFKRTV
1M18– GVRTCEGCKGFFKRTVQKNAKYICL
GFFKRTVQKNAKYICLANKDCPVDK

B. Peptides identified by PSD inhibiting SMAC interaction with them

MAML2

SLTPSNFPSPNQSSRAFQGTDHSSD
2I8– PNQSSRAFQGTDHSSDLAFDFLSQQ
GTDHSSDLAFDFLSQQNDNMGPALN

BIRC2

QKMKYDFSCELYRMSTYSTFPAGVP
1E14 – ELYRMSTYSTFPAGVPVSERSLARA
TFPAGVPVSERSLARAGFYYTGVND

ARNT

TLYDQVHPDDVDKLREQLSTSENAL
2J14– DVDKLREQLSTSENALTGRILDLKT
STSENALTGRILDLKTGTVKKEGQQ

Figure S4. Peptides sequences directly interact with PSD (A) or PSD prevented their interaction with SMAC (B)
The interacting peptide sequence red color and the peptides located before and after it in black are presented, with the spot number and their protein of origin are also presented.

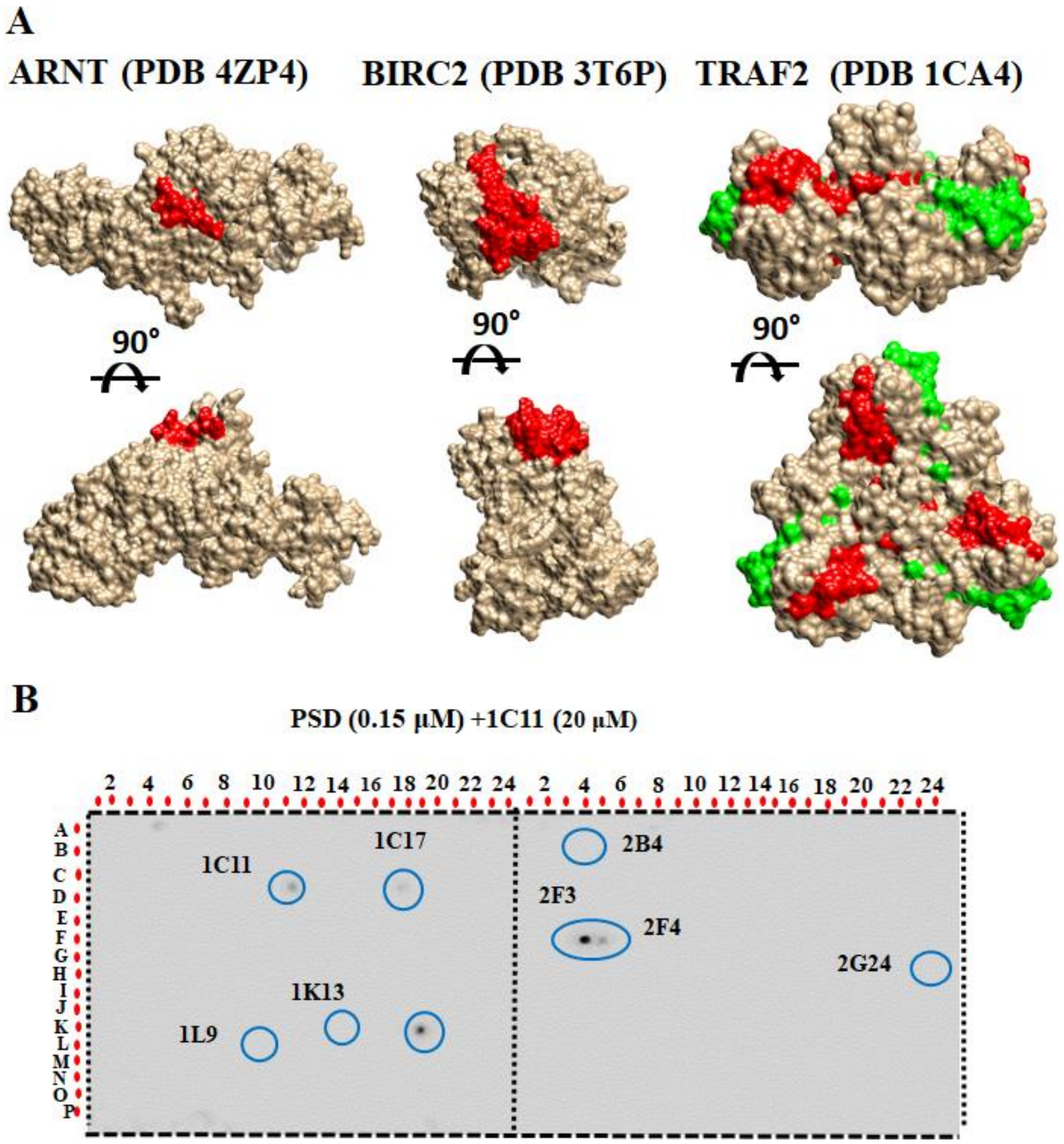

Figure S5. Peptides identified from direct interact with PSD or PSD prevented their interaction with SMAC labeled in the proteins they derived from

A. Positions of peptides in space-filling models of ARNT (PDB_ID 4ZP4), peptide 2J14 (red spheres), BIRC2, (PDB_ID 3T6P) peptide 1R14 (red spheres) and TRAF2 (PDB_ID 1Ca4) 1K13 (red spheres) and 1K18 (green spheres). All were prepared with UCSF Chimera [31]. **B.** Glass-bound peptide array consisting of overlapping peptides derived from 15 SMAC/PSD-interacting proteins was incubated overnight with PSD (0.15 µM) pre-incubated with its interacting peptide representing spot 1C11, followed by array blotting with anti-PSD antibodies. The peptide spots in the array that 1C11 peptide prevented or highly decreased their interaction with PSD are circled. This blotting was done in parallel with the control (PSD alone) shown in Fig. 6E.

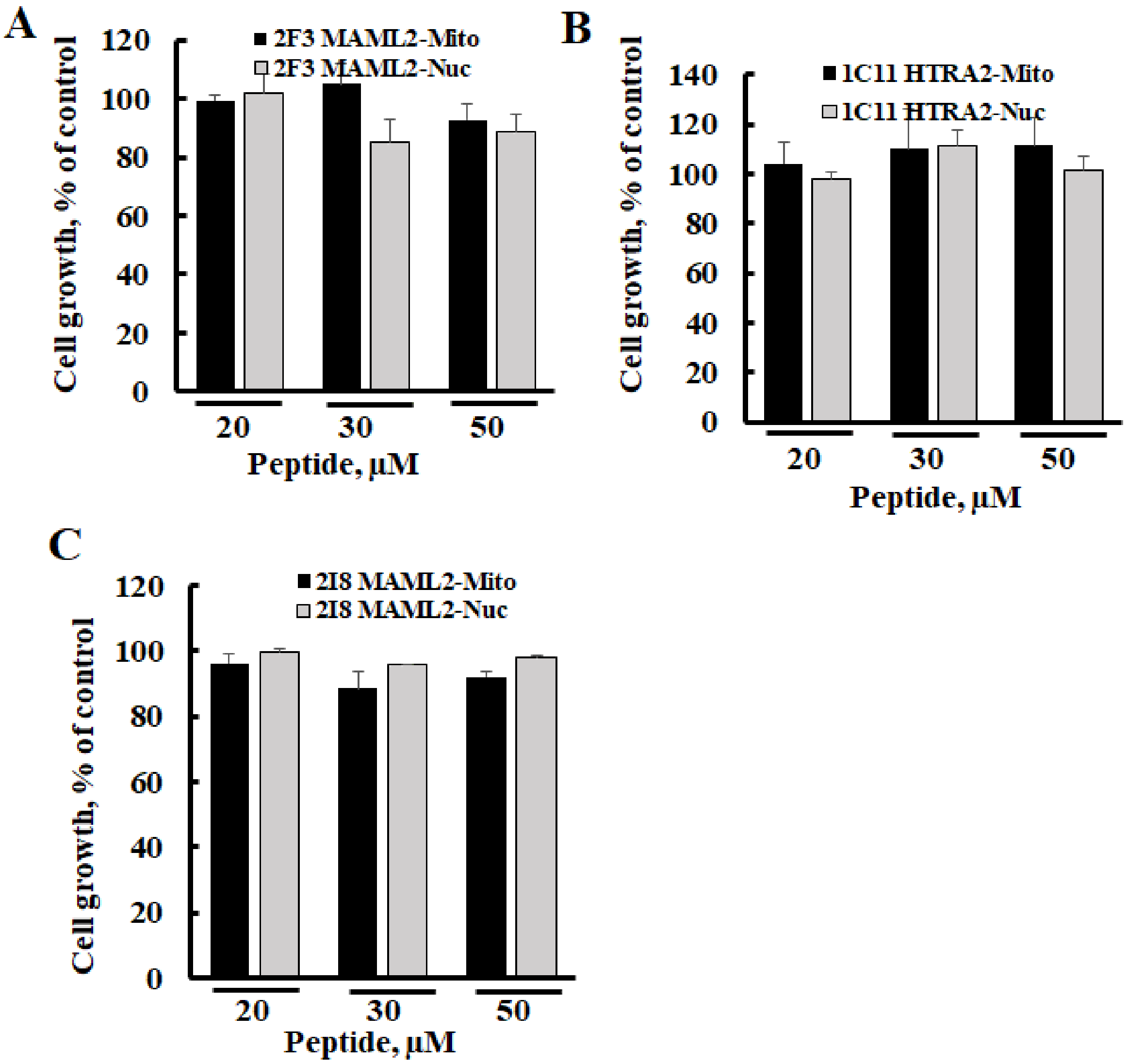

Figure S6. PSD-interacting peptides do not inhibit cell growth of epithelial HaCaT cells
(**A-C**) Cell growth inhibition following incubation of HaCat cells with the indicated mitochondria- (black bars) or nucleus-targeted peptides (grey bars) for 24h in a serum-free medium, and cell proliferation was assayed using the SRB method. Results are the means ± SEM (n=3).

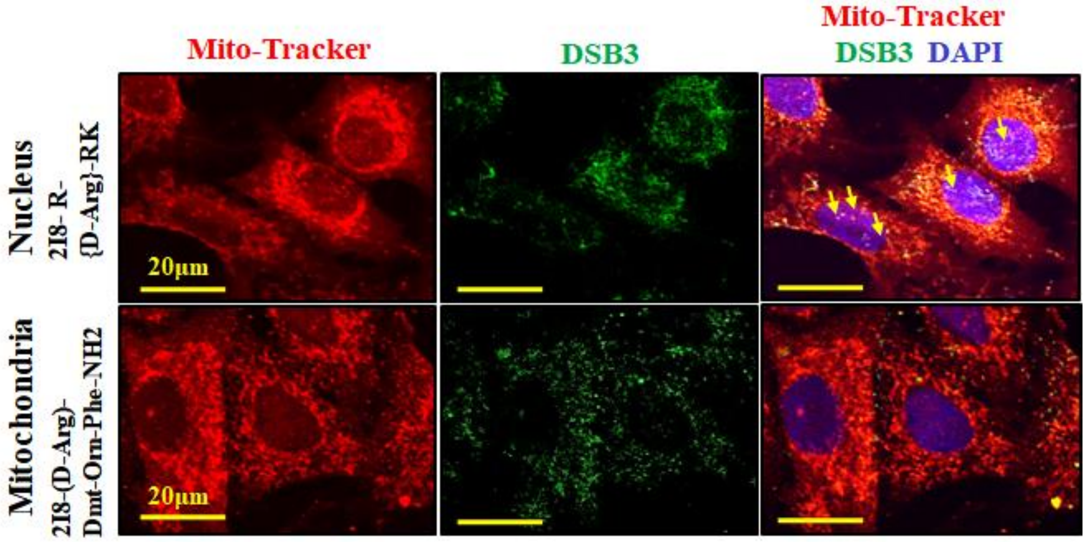

Figure S7. 2I8 peptide targeted to the nucleus or to the mitochondria reaches these compartments

A549 cells were incubated for 90 min with 5 μM FITC-labeled mitochondria- or nucleus-targeted peptide, were stained with mitotracker (250nM) for 45 min followed by incubation with DSB-3 (10 μM) for 2h, nuclei were stained with DAPI and visualized by confocal microscope for subcellular localization. Arrows point peptide presence in the nucleus. The nucleus-targeted peptide also presence in the mitochondria, probable due to its highly positive charge ad possible accumulation in the mitochondria due to the membrane potential. Scale bar represents 20 μm.

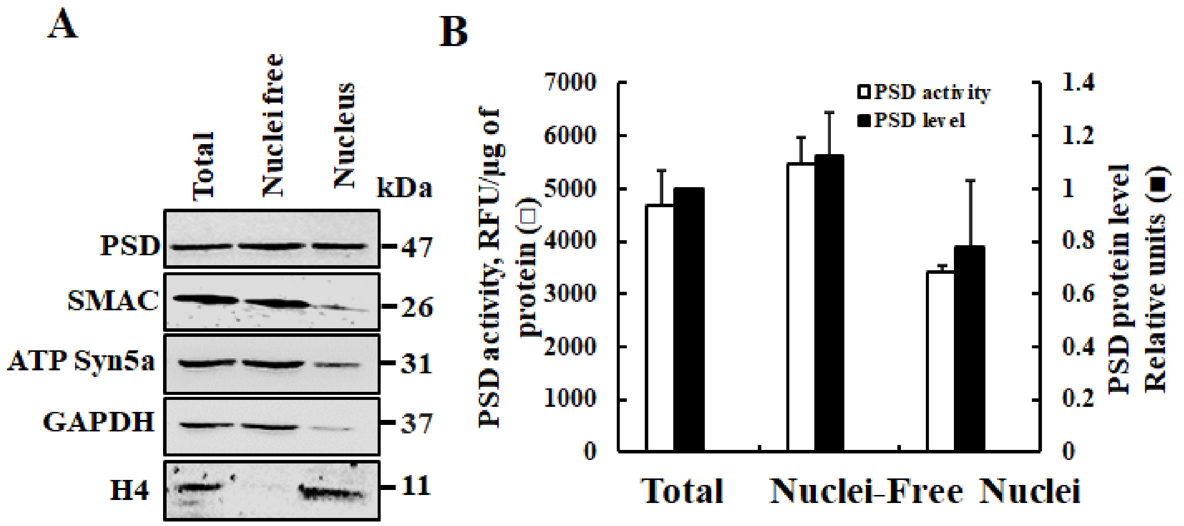

Figure S8. PSD is present in cell nucleus

(A) For PSD and SMAC sub-cellular localization in cells, nuclear extracts were prepared from NCI-H-1563 cells using a nuclear/cytosol fractionation kit (Biovision, Milpitas, CA), following the manufacturer's instructions. Before centrifugation (Total) and after centrifugation (16,000 g, 10 min), the supernatant (cytosolic fraction), and pellet (nuclear fraction) were re-suspended in the original volume and subjected to immunoblotting for SMAC, PSD, GAPDH (cytosolic), ATPsyn5a (mitochondria) and histone 4 (H4, nuclear fraction). **(B)** The total, nuclear-free and nuclear fractions were subjected to immunoblotting and quantitative analysis of the PSD levels (black bars) and to PSD activity assay (white bars) as described in the Materials and Methods section**.** Results represent the means ± SEM (n=3).
